# Development of Diversified Methods for Chemical Modification of the 5,6-Double Bond of Uracil Derivatives Depending on Active Methylene Compounds

**DOI:** 10.3390/molecules17066519

**Published:** 2012-05-30

**Authors:** Hironao Sajiki, Yusuke Iida, Kanoko (Yasunaga) Ikawa, Yoshinari Sawama, Yasunari Monguchi, Yukio Kitade, Yoshifumi Maki, Hideo Inoue, Kosaku Hirota

**Affiliations:** 1 Laboratory of Organic Chemistry, Gifu Pharmaceutical University, 1-25-4 Daigaku-nishi, Gifu 501-1196, Japan; 2 Graduate School of Engineering, Osaka City University, 3-3-138 Sugimoto, Sumiyoshi-ku, Osaka 558-8585, Japan

**Keywords:** C-C bond formation, active methylene, carbanion, uracil, uridine

## Abstract

The reaction of 5-halogenouracil and uridine derivatives **1** and **7** with active methylene compounds under basic conditions produced diverse and selective C-C bond formation products by virtue of the nature of the carbanions. Three different types of reactions such as the regioselective C-C bond formation at the 5- and 6-positions of uracil and uridine derivatives (products **2**, **5**, **8**, **17**, **20** and **21**), and the formation of fused heterocycle derivatives 2,4-diazabicyclo[4.1.0]heptane (**15**) and 2,4-diazabicyclo-[4.1.0]nonane (**16**) via dual C-C bond formations at both the 5- and 6-positions were due to the different active methylene compounds used as reagents.

## 1. Introduction

Many 5-substituted pyrimidine nucleoside derivatives and their base moieties possess antimicrobial, antifungus, antivirus and anticancer activities due mainly to antimetabolic effects (Table 1) [1,2,3,4,5,6]. Specifically, 5-fluorouracil (5-Fu) is a widely-used anticancer drug [1,2,3,4,5] and 5-fluoro-2'-deoxyuridine (floxuridine) and 5'-deoxy-5-fluorouridine (doxifluidine) are also well known as cancer drugs especially effective in the treatment of kidney carcinoma [1,2,3,4,5,6] and digestive system cancer, respectively. 5-Iodo-2'-deoxyuridine (idoxuridine) is an effective drug for herpes simplex virus mainly used as eye-drops [7], and 5-bromo-2'-deoxyuridine (bioxuridine) is used as a radiation enhancer. Furthermore, zidovudine and sanilvudine exhibit reverse transcriptase inhibitory activity and are in widespread clinical use as anti-HIV drugs. Since most of the biologically-active chemically modified pyrimidine derivatives are functionalized on the 5,6-double bond (especially the 5-position), the development of easy-to-use and specific chemical modification methods at the 5 and 6-positions of the pyrimidine nuclei is still quite important as related to the creation of novel antimetabolites.

**Table 1:** Selected biologically active 5-substituted pyrimidine nucleoside derivatives.

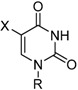	**R**	**X**	**Generic name**
	H	F	Fluorouracil (5-Fu)
	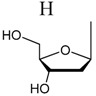	F	Floxuridine
		I	Idoxuridine
		Br	Broxuridine
	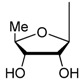	F	Doxifluridine
	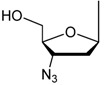	Me	Zidovuridine
	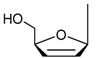	Me	Sanilvudine

**Scheme 1:**
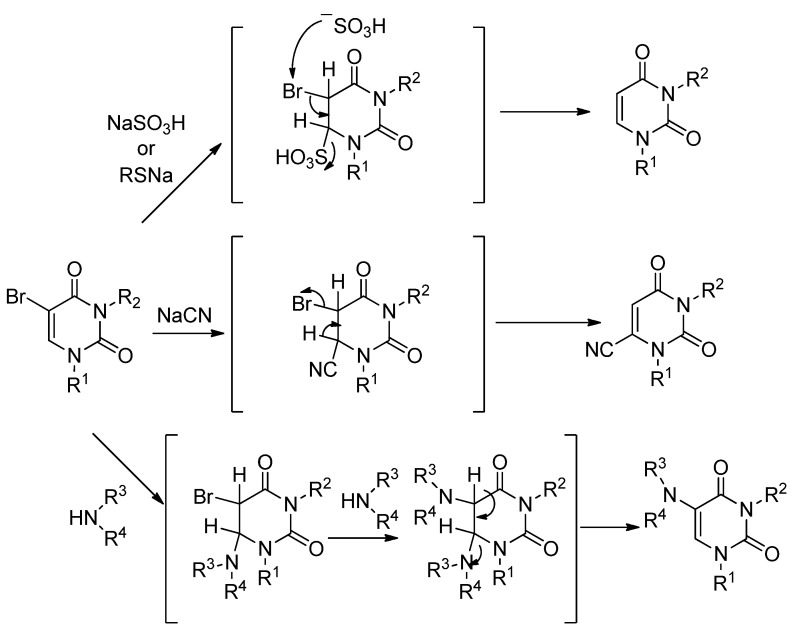
Formation of 5-substituted uracil and uridine derivatives.

A number of chemical modification methods at the 5-position of the uracil derivatives have been reported in the literature [8], although the functionalization methods for the 6-position have rarely been reported. Pd-catalyzed C–C bond formation reactions at the 5-position of the uracil ring, such as the Heck [9], Stille [10], and Sonogashira reactions [11,12], *etc*. [13,14] have been investigated in detail as widely applicable methods. The Mannich reaction [15,16,17,18], hydroxymethylation [19,20], the Morita-Baylis-Hillman reaction [21,22] and Wittig reaction using 5-hydroxyuridine [23] are also useful methods to synthesize chemically-modified uracil derivatives possessing a carbon functional group at the 5-position. Furthermore, it is well known that 5-bromouracils react easily with several nucleophiles [24] (Scheme 1). 

Simple debromination smoothly occurred by the use of sulfite or thiolate anion (^−^SO_3_H or ^−^SR) [24,25,26,27,28,29], and the cyanide anion (^−^CN) induced the *cine*-substitution reaction to produce 6-cyanouracil derivatives [28,30,31,32]. The reaction with amines produced 5-substituted uracil derivatives [28]. It is well known that all these reactions occurred via the 5,6-dihydro adducts as intermediates. On the other hand, only few carbanion-mediated nucleophilic reactions of the 5-bromo-uracil derivatives have been reported. Among them, Inoue *et al.* pioneeringly reported the formation of the 5-bis(ethoxycarbonyl)methyl-substituted uridine derivative by the reaction of 5-bromo-5',*N*_3_-dibenzoyl-2',3'-*O*-isopropylideneuridine and the carbanion generated from dimethylmalonate and 1,8-diazabicyclo[5,4,0]undec-7-ene (DBU) [33]. It has been proven that the 5-bis(ethoxycarbonyl)methyl-substituted uridine derivative was generated via the 5,6-di-bis(ethoxycarbonyl)methyl-substituted 5,6-dihydrouridine intermediate in our preliminary research as a collaboration project with Inoue *et al.* [34]. We now provide detailed results for the formation of the 5-substituted-uracil derivatives starting from the 5-halogenouracil derivatives using the carbanion generated from active methylene compounds and bases. The diversity of the reaction of the 5-bromouracil derivatives with carbanions exclusively based on the kind of active methylene compounds as carbanion sources.

## 2. Results and Discussion

Our initial studies focused on the formation of the 5-substituted uracils. 5-Bis(ethoxycarbonyl)-methyl-1,3-dimethyluracil (**2**) was obtained by the reaction of 1,3-dimethyl-5-halogenouracils (**1**) and diethyl malonate (3.3 equiv.) together with sodium ethoxide (3.0 equiv., generated*in situ* from sodium metal and anhydrous EtOH) in anhydrous EtOH at rt in 60–67% isolated yields (Table 2).

While **2** gave satisfactory spectral and microanalytical results consistent with the chemical structure, it was transformed into the known compound 1,3-dimethyluracil-5-acetic acid (**3**) [23], in a refluxing 47% HBr aqueous solution for 1 h in 95% yield to further confirm the structure. The results in Table 2 show that the rate of the reaction can be significantly affected by the type of halogens (Br, Cl and F). It is noteworthy that the reaction smoothly proceeded by the use of even 5-fluoro-1,3-dimethyluracil (**1c**) as the substrate although a prolonged reaction time was necessary (Entry 3).

**Table 2:** Formation of 5-bis(ethoxycarbonyl)methyl-1,3-dimethyluracil (**2**).

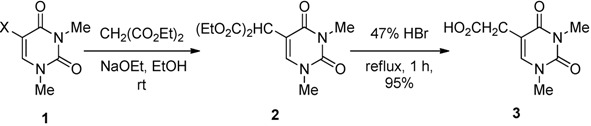				
Entry	Starting Compd.	X	Reaction time (h)	Yield (%)
1	1a	Br	8	60
2	1b	Cl	16	67
3	1c	F	24	65

Although the highly stable C-F bond at the 5-position of the uracil ring severely retards the substitution between the F atom and malonate carbanion, there is a significant acceleration effect by the strongly electron-withdrawing nature of the F atom to reduce the electron density at the conjugated 6-position of the uracil ring, which suggests that the carbon at the 6-position undergoes a decrease in electron density compared to Br and Cl. A balance of these opposite properties seems to influence the reactivity of the 5-fluoro-1,3-dimethyluracil (**1c**) in a subtle way.

**Scheme 2:**
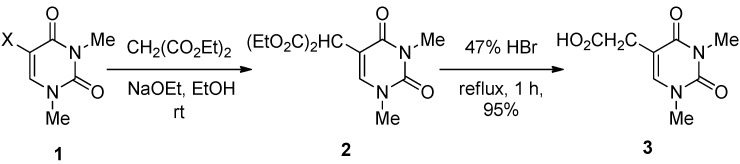
Formation of 5,6-di-[bis(ethoxycarbonyl)methyl]-5,6-dihydro-1,3-dimethyl-uracil (**4**) and its reactivity.

In relation to these results, we detected the presence of an intermediate **4** during the reaction of **1a** and diethyl malonate carbanion by TLC analysis (invisible under a UV-lamp, but the spot was directly stained by iodine absorption). The intermediate **4** was isolated in 41% yield along with 33% of unchanged **1a** by interruption of the reaction after a short time (2 h). The structure of the intermediate **4** was assigned by spectral and microanalytical results to 5,6-di-[bis(ethoxycarbonyl)methyl]-5,6-dihydro-1,3-dimethyluracil with a *trans*-configuration based on the ^1^H-NMR spectral data. The isolated intermediate **4** could be transformed by only stirring with sodium ethoxide in anhydrous EtOH at rt for 8 h to give the corresponding 5-bis(ethoxycarbonyl)methyl-1,3-dimethyluracil (**2**) in 67% yield (Scheme 2).

**Scheme 3:**
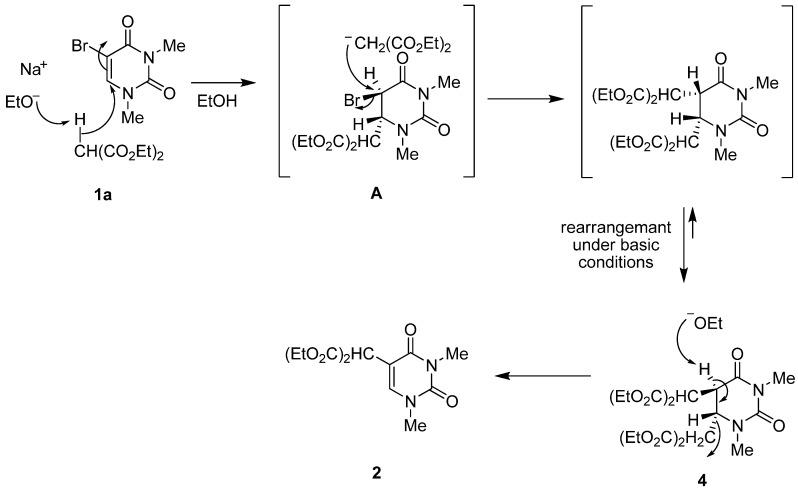
Plausible reaction mechanism.

Based on these results, the plausible reaction mechanism for the formation of **2** involving a Michael 1,4-addition is indicated in Scheme 3. The intermediate **4** could be obtained via the generation of the C-6 malonate adduct (**A**) by the nucleophilic attack of a diethyl malonate carbanion on the 6-position of the uracil ring and nucleophilic substitution between the bromine atom at the 5-position (*sp*^3^-carbon) of **A** and the remaining malonate carbanion (SN_2_ reaction). Subsequent C–C bond cleavage at the 6-position of **4** under basic conditions (E_2_ reaction) is a key and rate-determing step for the formation of **2**, and this is why the 5,6-di-[bis(ethoxycarbonyl)methyl]-5,6-dihydro-1,3-dimethyluracil (**4**) could be isolated (Scheme 3) [24].

**Scheme 4:**
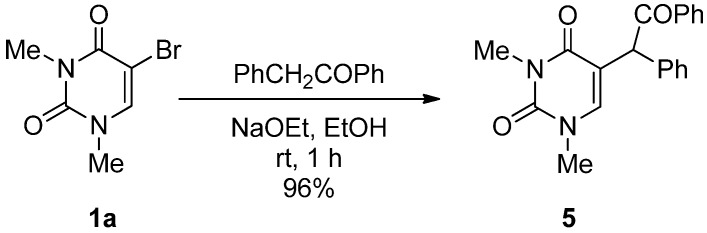
Formation of 5-(α-benzoyl)benzyl-1,3-dimethyluracil (**5**).

The C–C bond formation reaction at the 5-position of **1a** was also achieved using benzylphenylketone as a carbanion source, and 5-(α-benzoyl)benzyl-1,3-dimethyluracil (**5**) was obtained in 96% yield after only a 1 h reaction at rt (Scheme 4).

**Scheme 5:**
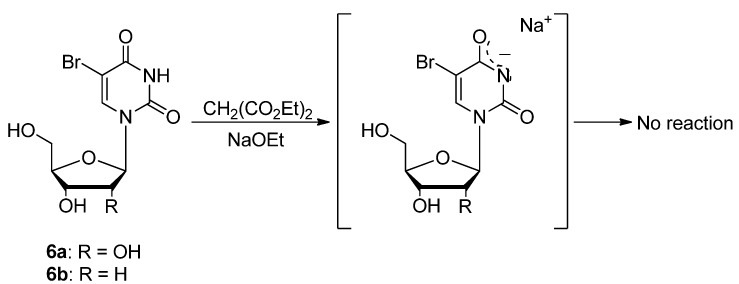
Reactivity of *N^3^*-non-protected 5-bromouridines.

Since these reactions using active methylene compounds as nucleophile sources were essential to perform under strong basic conditions due to the generation of carbanions, the use of the unprotected 5-bromouridine (**6a**) or 5-bromo-2'-deoxyuridine (**6b**) at the 3-position as a substrate were not suitable due to the formation of the inactive uracil-anion (Scheme 5).

**Scheme 6:**
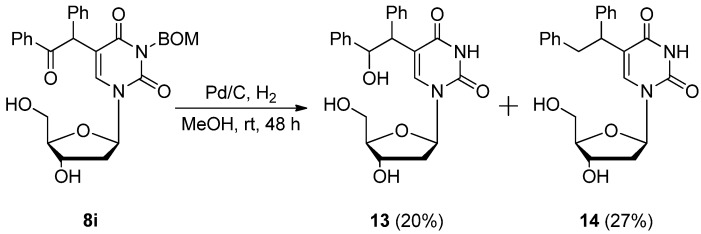
Deprotection of 5-(α-benzoyl)benzyl-3-benzyloxymethyl-2'-deoxyuridine (**8i**) under Pd/C-catalyzed hydrogenation conditions.

**Scheme 7:**
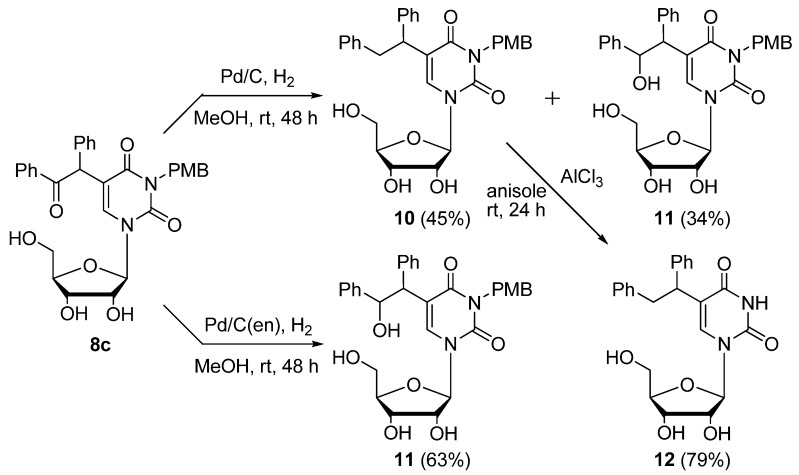
Pd/C- or Pd/C(en)-catalyzed hydrogenation of 5-(α-benzoyl)benzyl-3-methoxybenzyluridine (**8c**).

Hence, we next investigated the adding of a protective group to the 3-position of the uridine derivatives. 5-Bromo-3-*p*-methoxybenzyluridine (**7a**) was smoothly reacted with dimethyl malonate and benzylphenylketone at rt and the corresponding 5-substituted products **8a**–**c** were obtained in moderate yields (Table 3, Entries 1–3). These products were easily deprotected to give **9a**–**c** by stirring with AlCl_3_ (8.0 equiv.) in anisole at rt [35]. Since this deprotection method was unfortunately not applicable to the deprotection of the 3-*p*-methoxybenzyl-2'-deoxyuridine derivatives due to the instability of the *N*-glycosidic bond, we next investigated the introduction of the benzyloxymethyl (BOM) functionality as an *N^3^*-protective group [36,37]. The C–C bond formation reaction at the 5-position of the both 5-bromo-*N^3^*-BOM protected uridine (**7b**) and deoxyuridine (**7c**) easily proceeded by the use of diethyl and dimethyl malonates and benzylphenylketone, respectively, to give the 5-substituted uridine derivatives **8d**–**i** in 42–52% yields (Entries 4–9). Furthermore, the *N*_3_-BOM protective group of the 5-bis(ethoxycarbonyl)methyl-substituted uridines (compounds **8d**,**e**,**g**,**h**) was deprotected under Pd/C-catalyzed hydrogenation conditions in MeOH at rt in good to moderate yields (Entries 4, 5, 7 and 8). Although the deprotection took a prolonged reaction time (72 h), it was fortunately revealed that the addition of ammonium acetate (NH_4_OAc, 1.0 equiv.) could efficiently enhance the deprotection of **8g** and **8h**, and the deprotected products **9d** and **9e** were obtained in better yields (72 and 86%, respectively) within shorter reaction periods (20 and 24 h, respectively, Entries 7 and 8). While the deprotection intricately proceeded in the case of the 5-(α-benzoyl)benzyl-3-benzyloxymethyluridine derivatives **8f**,**i** due to the existence of an additional reducible functionality (aromatic ketone, Entries 6 and 9, Scheme 6), 5-(2-hydroxy-1,2-diphenylethyl)-2'-deoxyuridine (**13**, 20% yield) and 5-(1,2-diphenylethyl)-2'-deoxyuridine (**14**, 27%) could be isolated by careful preparative TLC (eluent: 5:1 CHCl_3_–MeOH). Inspired by these results, we attempted the functional group transformation under Pd/C-catalyzed hydrogenation conditions using 5-(α-benzoyl)benzyl-3-methoxybenzyluridine (**8c**) as a substrate. Consequently, a mixture of 3-*p*-methoxybenzyl-5-(1,2-diphenylethyl)uridine (**10**, 45% yield) and 3-*p*-methoxybenzyl-5-(2-hydroxy-1,2-diphenylethyl)uridine (**11**, 34% yield) was obtained and **10** was smoothly deprotected by the treatment with AlCl_3_ at rt for 24 h to give the corresponding 5-(1,2-diphenylethyl)uridine (**12**) in 79% yield. On the other hand, when the Pd/C-ethylenediamine complex [Pd/C(en)][38,39,40,41,42,43,44,45,46] was used as a catalyst, the chemoselective hydrogenation occurred and **11** was isolated as the sole product (Scheme 7).

**Scheme 8:**
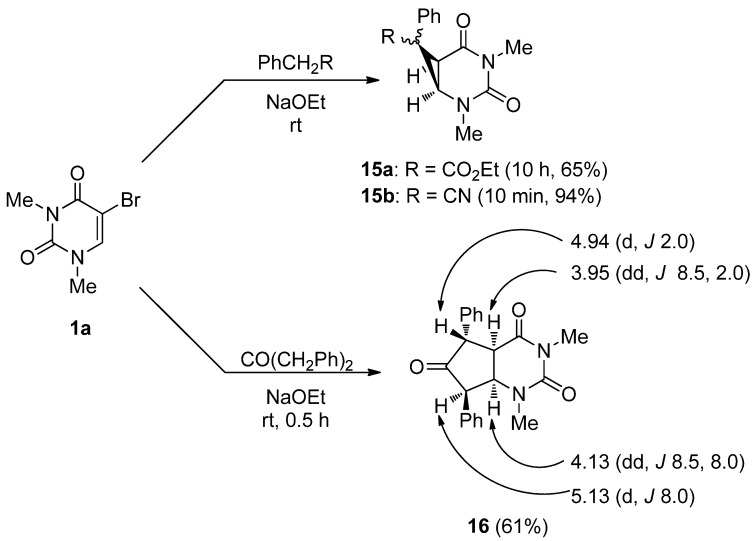
Formation of 2,4-diazabicyclo[4.1.0]heptane (cyclopropane) and 2,4-diazabicyclo[4.1.0]nonane (cyclopentane) derivatives.

Next we investigated the reaction of **1a** with ethyl phenylacetate and benzyl cyanide in the presence of NaOEt as a base at rt. Surprisingly, and against all expectation, the 2,4-diazabicyclo[4.1.0]heptane derivatives **15a**,**b** were obtained as the sole product (Scheme 8). The reaction was also found to proceed by the use of DBU instead of NaOEt. The structures of products **15a** and **15b** were supported by the spectral data and microanalytical results, and the characteristic AB pattern peaks of the bridgehead protons [**15a**: δ 3.20 (C-6–H), 3.72 (C-1–H), *J*_1,6_ = 9.0 Hz; 15b: δ 3.03 (C-6–H), 3.58 (C-1–H), J1,6 = 8.5 Hz] as a 2,4-diazabicyclo[4.1.0]heptane nucleus were also clearly observed in the ^1^H-NMR spectrum [47]. Although the 2,4-diazabicyclo[4.1.0]heptane derivatives were likely the intermediates for the generation of the 5-substituted uracils [33,34], **15a** was quite stable under basic conditions (NaOEt), even at the reflux temperature of EtOH.

**Table 3:** Preparation of 5-substituted uridine derivatives.

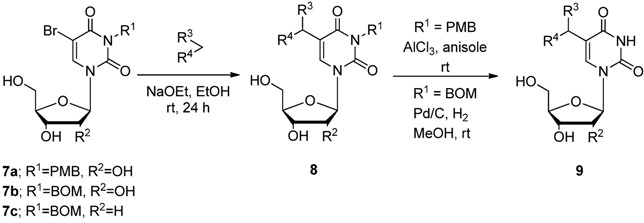										
Entry	Starting Compd. (7)			R^3^	R^4^	Product (8)	Yield (%)	Product (9)	Time (h)	Yield (%)
		R^1^	R^2^							
1	7a	PMB	OH	CO_2_Et	CO_2_Et	8a	51	9a	12	46
2	7a	PMB	OH	CO_2_Me	CO_2_Me	8b	47 ^a^	9b	24	42
3	7a	PMB	OH	Ph	COPh	8c	60	9c	4	82
4	7b	BOM	OH	CO_2_Et	CO_2_Et	8d	52	9a	72	69
5	7b	BOM	OH	CO_2_Me	CO_2_Me	8e	47 ^a^	9b	72	67
6	7b	BOM	OH	Ph	COPh	8f	47	9c	–	– ^b^
7	7c	BOM	H	CO_2_Et	CO_2_Et	8g	48	9d	72 (20) ^c^	64 (72) ^c^
8	7c	BOM	H	CO_2_Me	CO_2_Me	8h	42 ^a^	9e	72 (24) ^c^	59 (86) ^c^
9	7c	BOM	H	Ph	COPh	8i	43	9f	–	–^b,d^

^a^ The reaction was performed using NaOMe in anhydrous MeOH; ^b^ Complex mixture; ^c^ With NH_4_OAc; ^d^ See Scheme 7.

**Scheme 9:**
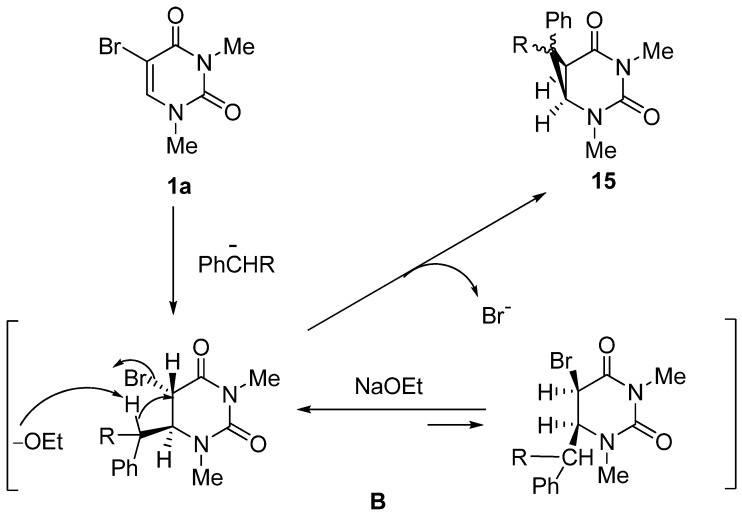
Plausible reaction sequence for formation of 2,4-diazabicyclo[4.1.0]heptanes.

The reaction sequence for the formation of the 2,4-diazabicyclo[4.1.0]heptanes is proposed to occur as shown in Scheme 9. An initial nucleophilic attack at the 6-position of **1a** by the carbanion could produce the C-6 adduct (**B**). The subsequent intramolecular cyclization between the C-5 position and active methine moiety of **B** accompanied by elimination of the bromide anion from the *sp*^3^ C-5 position results in the formation of the fused cyclopropane derivatives (**15**).

Furthermore, 5-bromo-1,3-dimethyluracil (**1a**) was easily transformed into the corresponding 2,4-diazabicyclo[4.1.0]nonane derivative **16** under similar reaction conditions by the use of dibenzylketone as a 1,3-ambident active methylene compound. While there could be eight diastereoisomers due to the four asymmetric carbons of **16**, the ^1^H-NMR analysis suggested that **16** is a single diastereomer possessing a *trans-cis-cis* configuration. 

As noted earlier in the Introduction, not so many chemical modification methods at the 6-position of uracil derivatives are reported in the literature, including the formation of 6-cyanouracil derivatives due to the *cine*-substitution reaction as shown in Scheme 1 [28,30,31,32]. In other examples, Tanaka and Miyasaka *et al.* reported the electrophilic functionalization at the 6-position of 2',3'-isopropylidene-5'-methoxymethyluridine via lithiation at the 6-position of the uracil ring [48], and the photochemically-induced nucleophilic substitution at the 6-position of 6-iodo-2'3'-isopropylidene-5'-methoxymethyl-uridine [49]. Needless to say, the normal nucleophilic substitution of the 6-halogenouracil derivatives under basic conditions has also been reported in the literature [50].

**Scheme 10:**
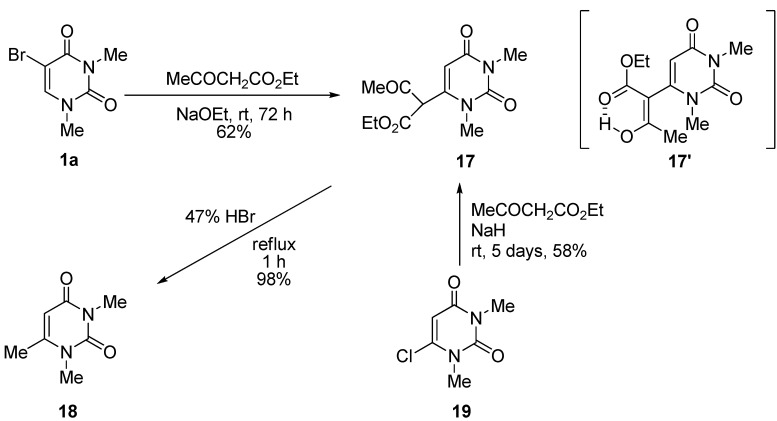
Formation of 6-substituted 1,3-dimethyluracils.

When **1a** was allowed to react with ethyl acetoacetate in the presence of NaOEt in anhydrous EtOH at rt for 72 h, the 1,3-dimethyluracil-6-(α-acetyl)acetic acid ethyl ester (**17**) was obtained in 62% yield together with 32% of the unchanged **1a** [Scheme 10; while the structure of **17** was indicated as the keto-form of the 6-substituent for the purpose of illustration, the actual structure in solution should be the enol-form with intramolecular hydrogen bond (**17**') based on ^1^H-NMR analytical data]. The reaction also proceeded by the use of NaH as a base in anhydrous DMF. The structure of **17** was supported on the basis of the spectral data and microanalytical results, and confirmed by comparison of the spectral data with those of the product of the alternative synthesis based on the reaction using 6-chloro-1,3-dimethyluracil (**19**) and ethyl acetoacetate in the presence of NaH in anhydrous DMF at rt. Furthermore, **17** could be quantitatively converted into the well-known 1,3,6-trimethyluracil (**18**) in 98% yield after a 1 h reflux in a 47% HBr solution.

**Scheme 11:**
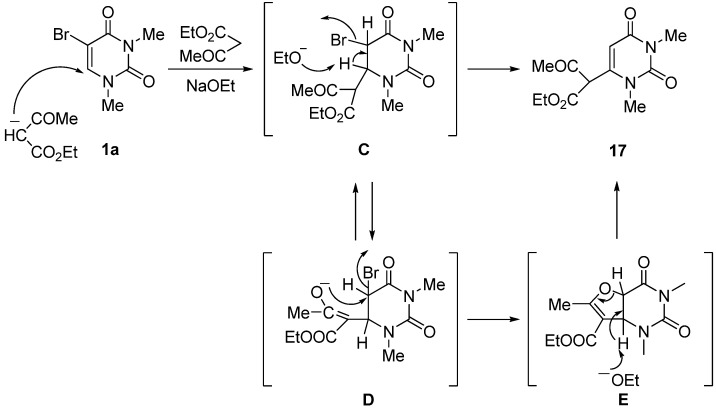
Plausible reaction mechanism for the formation of 6-substituted product (**17**).

The reaction sequence for the formation of **17** is outlined in Scheme 11. Although the Michael adduct (**C**) can be normally converted to the 6-substituted product **17** accompanied by the elimination of HBr (*cine*-substitution), it is rather reasonable to suggest that the reaction proceeded via the cyclic intermediate (**E**) generated by the intramolecular nucleophilic attack of the corresponding enolate anion of **D** on the 5-position under strong basic conditions [30,31,32]. We believe that is why the formation of the 6-substituted product **17** occurs when using ethyl acetoacetate.

**Scheme 12:**
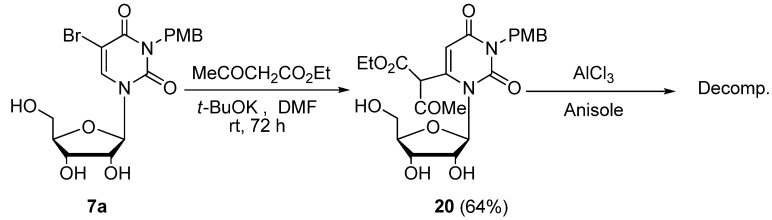
Preparation of the 3-*p*-methoxybenzyluridine-6-(α-acetyl)acetic acid ethyl ester (**20**).

**Scheme 13:**
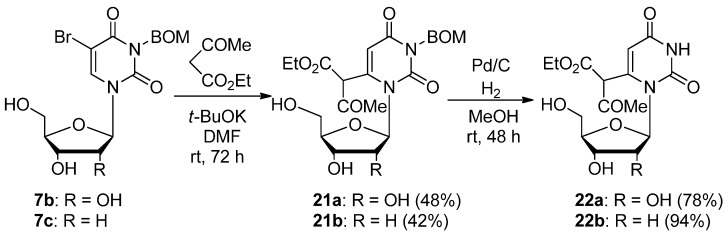
Preparation of the 3-benzyloxymethyluridine 6-(α-acetyl)acetic acid ethyl ester derivatives **21a** and **21b** and their efficient deprotection.

The present reaction is applicable for the preparation of the 3-*p*-methoxybenzyluridine-6-(α-acetyl)acetic acid ethyl ester (**20**). The treatment of 5-bromo-3-*p*-methoxybenzyluridine (**7a**) with ethyl acetoacetate (3.3 equiv.) and *t*-BuOK (3.0 equiv.) in DMF at rt for 72 h gave **20** in 64% yield (Scheme 12), while the deprotection of **20** using AlCl_3_ [35] was unsuccessful due to decomposition. Upon treatment of 3-benzyloxymethyl-5-bromouridine (**7b**) and 3-benzyloxymethyl-5-bromodeoxyuridine (**7c**) with ethyl acetoacetate under analogous conditions, the corresponding 3-benzyloxymethyluridine-6-(α-acetyl)acetic acid ethyl ester derivatives **21a**,**b** were obtained in 48 and 42% yields, respectively. These products **21a**,**b** were easily deprotected to **22a** and **22b** (while the structures of **21/22a**,**b** were indicated as the keto-forms of the 6-substituent for the purpose of illustration, the actual structure in solution should be the enol-forms with intramolecular hydrogen bond based on centering on the ^1^H-NMR data) under neutral Pd/C-catalyzed hydrogenation conditions in MeOH at rt (Scheme 13).

These diversified reactivities by virtue of the difference in the active methylene compounds may be controlled by the p*K*_a_ values of the particular active methylene compounds. 5-Substituted products (*i.e*., compounds **2**, **5** and **8**) are obtained when using diethyl- and dimethyl malonate and benzylphenyl-ketone possessing moderate acidities (p*K*_a_ in DMSO: 16.4 [51], 15.9 [52] and 17.7 [53], respectively) due to the preference of the intermolecular nucleophilic attack at the 5-position of the adduct (**A**, Scheme 3) because of the comparatively longer life of the corresponding carbanions. On the other hand, cyclopropane and cyclopentane derivatives **15** and **16** are produced when using the less acidic active methylene compounds, such as ethyl phenylacetate, benzyl cyanide and dibenzylketone (p*K*_a_ in DMSO: 22.7 [54], 21.9 [55] and 18.7 [53], respectively), due to the intramolecular nucleophilic attack at the 5-position of **B** (Scheme 9) in preference to the intermolecular nucleophilic attack based on excessive amounts of the active methylene compounds due to the unstable nature of the corresponding carbanions. In the case of ethyl acetoacetate (p*K*_a_ in DMSO: 14.2 [56]) capable of forming an enolate (**D**, Scheme 11) under basic conditions, a special reactivity was observed since the intramolecular cyclization by the enolate-attack at the 5-position preferentially proceeded to give the 6-substituted products **17**, **20** and **21**. However, an exact rational explanation is difficult because many other active methylene compounds are not applicable to present reactions by reason of the frequent occurrence of side-reactions such as polymerization and decomposition under basic conditions.

While the synthesized 5- and 6-substituted deoxyuridine derivatives **8g**–**8i**, **9g**, **9h**, **13**, **14**, **21b** and **22b** were evaluated for their antiviral activities against the herpes simplex virus (HSV), human cytomegalovirus (HCMV) and influenza A virus and cytostatic activity, these compounds unfortunately indicated no or minimal activities.

## 3. Experimental

### 3.1. General

All reagents were obtained from commercial sources and used without further purification. Analytical thin-layer chromatography (TLC) was carried out on pre-coated Silica gel 60 F-254 plates (32–63 µm particle size) and visualized with UV light (254 nm). The 10% Pd/C was obtained from Merck KGaA or Aldrich. Flash column chromatography was performed with Silica gel 60 (40–63 µm particle size, Merck KGaA) or Silica gel 60N (100–210 µm, Kanto Chemical). The ^1^H and ^13^C-NMR spectra were recorded by a JEOL AL 400 spectrometer (Tokyo, Japan), JEOL EX 400 spectrometer (400 MHz for ^1^H-NMR and 100 MHz for ^13^C-NMR) or JEOL TNG-GX270 spectrometer (270 MHz for ^1^H-NMR) with tetramethylsilane or residual protonated solvent used as a reference. Elemental analyses were carried out at the Microanalytical Laboratory of our university (YANACO CHN CORDER MT-5 instrument, Tokyo, Japan). The EI and FAB Mass spectra were obtained using a JEOL JMS-SX102A instrument (Tokyo, Japan). The UV spectra were obtained in ethanol using a Shimadzu UV-260 spectrophotometer (Kyoto, Japan). 

*5-Bis(ethoxycarbonyl)methyl-1,3-dimethyluracil* (**2**) (Table 2, Entries 1–3). (a) A solution of 5-bromo-1,3-dimethyluracil (**1a**) (1.85 g, 8.45 mmol) and diethyl malonate (4.48 g, 27.9 mmol) in ethanolic NaOEt [prepared from Na (584 mg, 25.4 mmol) in absolute EtOH (85 mL)] was stirred for 8 h at room temperature. The mixture was evaporated under reduced pressure and the residue was dissolved in H_2_O (20 mL). The mixture was neutralized with conc. HCl and extracted with CHCl_3_. The extract was dried over MgSO_4_ and the solvent was removed under reduced pressure. The residue was purified by column chromatography on silica gel with CHCl_3_ as the eluant to give compound **2** (1.51 g, 60%) as a colorless oil. ^1^H-NMR (CDCl_3_) 7.54 (1H, s, 6-H), 4.87 (s, 1H, CH), 4.21 (q, *J* = 7.0 Hz, 4H, CH_2_), 3.42 and 3.31 (each s, each 3H, NMe), 1.25 (t, *J* = 7.0 Hz, 6H, CMe); ^13^C-NMR (CDCl_3_) 167.9, 162.5, 151.2, 142.9, 105.6, 62.3, 48.0, 37.4, 28.3, 14.0; HRMS (EI) calcd. for C_13_H_18_N_2_O_6_ (M^+^): *m/z* 298.1165; found 298.1174; (b) A solution of 5-chloro-1,3-dimethyluracil (**1b**, 1.75 g, 10.0 mmol) and diethyl malonate (5.29 g, 33.0 mmol) in ethanolic NaOEt [prepared from Na (690 mg, 30.0 mmol) in absolute EtOH (100 mL)] was stirred for 16 h at room temperature and then treated as described above to give **2** (2.00 g, 67%); (c) A solution of 5-fluoro-1,3-dimethyluracil (**1c**, 474 mg, 3.00 mmol) and diethyl malonate (1.59 g, 9.90 mmol) in ethanolic NaOEt [prepared from Na (207 mg, 9.00 mmol) in absolute EtOH (30 mL)] was stirred for 24 h at room temperature and then treated as described above to give **2** (582 mg, 65%).

*1*,*3-Dimethyluracil-5-acetic acid* (**3**) [23]. A mixture of 5-bis(ethoxycarbonyl)methyl-1,3-dimethyluracil (**2**) (349 mg, 1.17 mmol) in a 47% HBr solution was refluxed for 1 h. The solvent was removed under reduced pressure, and the residue was purified by column chromatography on silica gel with CHCl_3_–MeOH (5:1) as the eluant to give **3** (220 mg, 95%), which was identical to the authentic sample.

*5*,*6-Di-[bis(ethoxycarbonyl)methyl]-5*,*6-dihydro-1,3-dimethyluracil* (**4**). A solution of 5-bromo-1,3-dimethyluracil (**1a**) (657 mg, 3.00 mmol) and diethyl malonate (1.59 g, 9.90 mmol) in ethanolic NaOEt [prepared from Na (207 mg, 9.00 mmol) in absolute EtOH (30 mL)] was stirred at room temperature for 2 h. The mixture was neutralized with Amberlite CG-50 (H^+^) and filtered. The ion exchange resin was washed with ethanol, and the combined filtrates were concentrated under reduced pressure. The residue was treated with H_2_O (30 mL). The aqueous solution was extracted with CHCl_3_ and the extract was dried over MgSO_4_ and concentrated *in vacuo*. The residue was purified by column chromatography on silica gel with benzene–EtOAc (6:1) as the eluant to give the starting material **1a** (217 mg, 33%) and the 5,6-dihydrouracil **4** (564 mg, 41%), which was recrystallized from EtOH. m.p. 85–86 °C; UV λ_max._ (EtOH) only end absorption; ^1^H-NMR (CDCl_3_) 4.29 (brd, *J* = 7.3 Hz, 1H, CH), 4.27–4.15 (m, 8H, CH_2_), 3.75 (d, *J* = 5.6 Hz, 1H, CH), 3.66 (d, *J* = 7.3 Hz, 1H, CH), 3.50 (brd, *J* = 5.6 Hz, 1H, CH), 2.96 and 3.16 (each s, each 3H, NMe), 1.35–1.20 (m, 12H, CMe); ^13^C-NMR (CDCl_3_) 168.3, 166.8, 166.8, 166.2, 166.1, and 152.4 (each C=O), 62.5, 62.3, 62.3, and 62.2 (each CH_2_), 55.6, 54.5, 52.0, and 44.6 (C-5, C-6 and CH × 2), 36.5 and 27.8 (each NMe), 13.9, 13.9, 13.9, and 13.8 (each CMe); MS (EI) *m/z* 459 (M^+^+H, 3%), 413 (25), 299 (100), 207 (47); Anal calcd. for C_20_H_30_N_2_O_10_: C, 52.39; H, 6.60; N, 6.11%; found: C, 52.14; H, 6.65; N, 6.11.

*Reaction of* **4** *with sodium ethoxide*: A solution of **4** (101 mg, 0.220 mmol) in ethanolic NaOEt [prepared from Na (14.9 mg, 0.650 mmol) in absolute EtOH (5 mL)] was stirred at room temperature for 8 h. The solvent was removed under reduced pressure and the residue was treated with H_2_O (20 mL). The solution was neutralized with c.HCl and the aqueous solution was extracted with CHCl_3_. The extract was dried over MgSO_4_ and concentrated *in vacuo*. The residue was purified by column chromatography on silica gel with benzene–EtOAc (6:1) as the eluant to give 1,3-dimethyluracil-5-malonic acid diethyl ester (**2**, 44.0 mg, 67%).

*5-(α-Benzoyl)benzyl-1*,*3-dimethyluracil* (**5**). A mixture of 5-bromo-1,3-dimethyluracil (**1a**) (1.32 g, 6.00 mmol) and benzylphenylketone (3.69 g, 19.8 mmol) in ethanolic NaOEt [prepared from Na (414 mg, 18.0 mmol) in absolute EtOH (60 mL)] was stirred at room temperature for 1 h. The solvent was removed under reduced pressure and the residue was treated with H_2_O (40 mL). The solution was neutralized with conc. HCl and the aqueous solution was extracted with CHCl_3_. The extract was dried over MgSO_4_ and concentrated *in vacuo*. The residue was purified by column chromatography on silica gel with CHCl_3_ as the eluant to give **5** (1.94 g, 96%), which was recrystallized from CHCl_3_. m.p. 128.5–130 °C; UV λ_max._ (EtOH) 249 nm (ε 16,300 dm^3^mol^–1^cm^–1^); ^1^H-NMR (CDCl_3_) 8.27–7.88 (m, 2H, COPh), 7.74–7.15 (m, 8H, Ph and COPh), 6.73 (brs, 1H, 6-H), 6.09 (brs, 1H, CH), 3.34 and 3.27 (each s, each 3H, NMe); ^13^C-NMR (CDCl_3_) 197.5, 163.2, 151.4, 142.1, 136.1, 135.3, 133.2, 129.5, 129.1, 128.9, 128.6, 128.0, 113.8, 50.8, 37.3, 28.1; MS (EI) *m/z* 334 (M^+^, 13%), 229 (100), 172 (25), 131 (38), 105 (70); Anal calcd. for C_20_H_18_N_2_O_3_: C, 71.84; H, 5.43; N, 8.38%; found: C, 71.79; H, 5.47; N, 8.37.

*5-Bromo-3-p-methoxybenzyluridine* (**7a**). *p*-Methoxybenzyl chloride (0.93 mL, 6.80 mmol) was added dropwise to a mixture of 5-bromouridine (2.00 g, 6.20 mmol) and K_2_CO_3_ (1.11 g, 8.05 mmol) in DMF (10 mL) at room temperature. The mixture was stirred for 24 h and filtered. The filtrate was concentrated *in vacuo*, and the residue was purified by recrystallization from MeOH to give **7a** (2.42 g, 88%) as a colorless powder. ^1^H-NMR (DMSO-*d*_6_) 8.60 (s, 1H, 6-H), 7.25 and 7.05 (each d, each *J* = 8.8 Hz, each 2H, C_6_H_4_), 5.75 (d, *J* = 4.9 Hz, 1H, 1'-H), 5.46 (brs, 1H, OH), 5.30 (brs, 1H, OH), 5.05 (brd, *J* = 5.9 Hz, 1H, OH), 4.93 (s, 2H, CH_2_), 4.03–3.99 (m, 2H, 2'-H and 3'-H), 3.87–3.82 (m, 1H, 4'-H), 3.77 (s, 3H, CH_3_), 3.62–3.57 (m, 2H, 5'-H); ^13^C-NMR (DMSO-*d*_6_) 158.5, 158.3, 149.9, 139.1, 129.5, 128.5, 113.7, 94.9, 89.7, 84.5, 73.9, 68.8, 59.7, 55.0, 44.0; MS (EI) *m/z* 442 (M^+^, 8%), 444 (8), 310 (25), 312 (25), 162 (12), 121 (100); HRMS (EI) calcd. for C_17_H_19_BrN_2_O_7_ (M^+^): 442.03756; found: 442.03681; Anal calcd. for C_17_H_19_BrN_2_O_7_: C, 46.07; H, 4.32; N, 6.32%; found: C, 45.96; H, 4.46; N, 6.33.

*5-Bromo-3-benzyloxymethyluridine* (**7b**). Benzyloxymethyl chloride (0.930 mL, 6.80 mmol) was dropwise added to a mixture of 5-bromouridine (2.00 g, 6.20 mmol) and K_2_CO_3_ (1.11 g, 8.05 mmol) in DMF (10 mL) at room temperature. The mixture was stirred for 24 h and filtered. The filtrate was concentrated *in vacuo*, and the residue was purified by column chromatography on silica gel with CHCl_3_-MeOH (50:1) as the eluant to give **7b** (1.51 g, 55%) as a colorless powder. ^1^H-NMR (DMSO-*d*_6_) 8.60 (s, 1H, 6-H), 7.34–7.25 (m, 5H, C_6_H_5_), 5.72 (d, *J* = 3.7 Hz, 1H, 1'-H), 5.50–5.47 (m, 1H, OH), 5.37–5.32 (m, 3H, OH and CH_2_), 5.09–5.05 (m, 1H, OH), 4.58 (s, 2H, CH_2_) 4.09–4.00 (m, 1H, 2'-H), 3.96–3.95 (m, 1H, 3'-H), 3.81–3.74 (m, 1H, 4'-H), 3.61–3.55 (m, 2H, 5'-H); ^13^C-NMR (DMSO-*d*_6_) 158.6, 150.0, 139.7, 128.1, 127.5, 127.4, 94.8, 89.7, 84.4, 74.0, 71.3, 71.1, 68.6, 59.6; MS (FAB, Gly) *m/z* 443 (M^+^+H, 8%), 445 (8%), 365 (5), 277 (12) 185 (100); HRMS (FAB, Gly) calcd. for C_17_H_19_BrN_2_O_7_ (M^+^): 442.0376; found: 442.0447; Anal calcd. for C_17_H_19_BrN_2_O_7_: C, 46.07; H, 4.32; N, 6.32; found: C, 46.07; H, 4.48; N, 6.28.

*5-Bromo-3-benzyloxymethyl-2'-deoxyuridine* (**7c**). Benzyloxymethyl chloride (0.460 mL, 2.59 mmol) was dropwise added to a mixture of 5-bromo-2'-deoxyuridine (1.00 g, 2.36 mmol) and K_2_CO_3_ (561 mg, 3.07 mmol) in DMF (10 mL) at room temperature. The mixture was stirred for 24 h and filtered. The filtrate was concentrated *in vacuo*, and the residue was purified by column chromatography on silica gel with CHCl_3_–MeOH (100:1) as the eluant to give **7c** (549 mg, 55%) as colorless oil. ^1^H-NMR (DMSO-*d*_6_) 8.49 (s, 1H, 6-H), 7.34-7.23 (m, 5H, C_6_H_5_), 6.10 (t, *J* = 6.3 Hz, 1H, 1'-H), 5.34 (s, 2H, CH_2_), 5.25 (brd, *J* = 4.4 Hz, 1H, OH), 5.21-5.18 (m, 1H, OH), 4.58 (s, 2H, CH_2_), 4.25-4.20 (m, 1H, 3'-H), 3.83-3.79 (m, 1H, 4'-H), 3.67-3.54 (m, 2H, 5'-H), 2.19-2.12 (m, 2H, 2'-H); MS (FAB, NBA) *m/z* 427 (M^+^+H, 5%), 429 (5), 154 (100), 146 (64); HRMS (FAB, NBA) calcd. for C_17_H_19_BrN_2_O_6_ (M^+^+H): 427.04265; found: 427.04935.

*5-Bis(ethoxycarbonyl)methyl-3-p-methoxybenzyluridine* (**8a**). A solution of 5-bromo-3-*p*-methoxybenzyluridine (**7a**) (500 mg, 1.13 mmol) and diethyl malonate (597 mg, 3.73 mmol) in ethanolic NaOEt [prepared from Na (77.8 mg, 3.38 mmol) in absolute EtOH (10 mL)] was stirred for 24 h at room temperature. The mixture was evaporated under reduced pressure, the residue was dissolved in H_2_O (30 mL), and the mixture was neutralized with NaHSO_4_. The solution was extracted with EtOAc and the extract was dried over MgSO_4_. The solvent was removed under reduced pressure, and the residue was purified by column chromatography on silica gel with CHCl_3_–MeOH (150:1) as the eluant to give **8a** (301 mg, 51%) as a colorless foam. ^1^H-NMR (DMSO-*d*_6_) 8.00 (s, 1H, 6-H), 7.26 and 6.88 (each d, each *J* = 8.7 Hz, each 2H, C_6_H_4_), 5.88 (d, *J* = 5.1 Hz, 1H, 1'-H), 5.46 (brd, *J* = 5.9 Hz, 1H, OH), 5.13–5.09 (m, 1H, OH), 4.94 (brd, *J* = 5.0 Hz, 1H, OH), 4.94 (s, 2H, CH_2_), 4.16 (q, *J* = 7.2 Hz, 4H, CH_2_ × 2), 4.03–3.98 (m, 1H, 2’-H), 3.95–3.90 (m, 1H, 3'-H), 3.90–3.89 (m, 1H, 4’-H), 3.74 (s, 3H, CH_3_), 3.62–3.45 (m, 2H, 5'-H), 1.19 (t, *J* = 7.1 Hz, 6H, CH_3_ × 2); ^13^C-NMR (DMSO-*d*_6_) 166.9, 161.1, 158.6, 150.2, 137.9, 129.5, 128.7, 113.6, 106.9, 88.9, 85.1, 73.8, 69.8, 61.5, 60.8, 55.0, 49.7, 43.3, 13.8; MS (FAB, NBA) *m/z* 523 (M^+^+H, 29%), 522 (7), 391 (9), 154 (100), 121 (76); HRMS (FAB, NBA) calcd. for C_24_H_31_N_2_O_11_ (M^+^+H) 523.185; found: 523.1935.

*5-Bis(methoxycarbonyl)methyl-3-p-methoxybenzyluridine* (**8b**). A solution of 5-bromo-3-*p*-methoxybenzyluridine (**7a**) (500 mg, 1.13 mmol) and dimethyl malonate (493 mg, 3.73 mmol) in ethanolic NaOEt [prepared from Na (77.8 mg, 3.38 mmol) in absolute EtOH (10 mL)] was stirred for 24 h at room temperature. The mixture was evaporated under reduced pressure, the residue was dissolved in H_2_O (30 mL), and the mixture was neutralized with NaHSO_4_. The solution was extracted with EtOAc and the extract was dried over MgSO_4_. The solvent was removed under reduced pressure and the residue was purified by column chromatography on silica gel with CHCl_3_–MeOH (150:1) as the eluant to give **8b** (262 mg, 47%) as a colorless foam. ^1^H-NMR (DMSO-*d*_6_) 7.99 (s, 1H, 6-H), 7.24 and 6.86 (each d, each *J* = 8.5 Hz, each 2H, C_6_H_4_), 5.84 (d, *J* = 3.9 Hz, 1H, 1'-H), 5.45 (brd, *J* = 5.8 Hz, 1H, OH), 5.13–5.11 (m, 1H, OH), 5.05 (brd, *J* = 4.3 Hz, 1H, OH), 4.85 (s, 2H, CH_2_), 4.64 (s, 1H, CH), 4.01–3.99 (m, 1H, 2'-H), 3.98–3.97 (m, 1H, 3'-H), 3.92–3.87 (m, 1H, 4'-H), 3.17 (s, 6H, CH_3_ × 2), 3.66 (s, 3H, CH_3_), 3.57–3.50 (m, 2H, 5'-H); MS (EI) *m/z* 494 (M^+^, 5%), 462 (17), 304 (18), 162 (17), 121 (100); HRMS (EI) calcd. for C_22_H_28_N_2_O_11_ (M^+^): 494.15366; found: 494.15423.

*5-(α-Benzoyl)benzyl-3-p-methoxybenzyluridine* (**8c**). A solution of 5-bromo-3-*p*-methoxybenzyluridine (**7a**) (500 mg, 1.13 mmol) and benzylphenylketone (732 mg, 3.73 mmol) in ethanolic NaOEt [prepared from Na (77.8 mg, 3.38 mmol) in absolute ethanol (10 mL)] was stirred for 24 h at room temperature. The mixture was evaporated under reduced pressure, the residue was dissolved in H_2_O (30 mL), and the mixture was neutralized with NaHSO_4_. The solution was extracted with EtOAc and the extract was dried over MgSO_4_. The solvent was removed under reduced pressure and the residue was purified by column chromatography on silica gel with CHCl_3_–MeOH (150:1) as the eluant to give **8c** (379 mg, 60%) as a colorless foam. ^1^H-NMR (DMSO-*d*_6_) 7.99 (d, *J* = 7.8 Hz, 2H, *o*-Bz), 7.55 (t, *J* = 7.3 Hz, 1H, *p*-Bz), 7.46 (t, *J* = 6.8 Hz, m-Bz), 7.35–7.31 (m, 5H, C_6_H_5_), 7.28 and 6.83 (each d, each *J* = 6.3 Hz, each 2H, C_6_H_4_), 7.01 and 7.04 (each s, total 1H, 6-H), 6.10 (s, 1H, CH), 5.79 (d, *J* = 5.4 Hz, 1H, 1'-H), 5.41 (brd, *J* = 5.9 Hz, 1H, OH), 5.33–5.32 (m, *J* = 5.8 Hz, 1H, OH), 5.10–5.06 (m, 1H, OH), 4.89 (s, 2H, CH_2_) 4.66–4.63 (m, 1H, 2'-H), 4.59–4.56 (m, 1H, 3'-H), 3.87–3.84 (m, 1H, 4'-H), 3.76 (s, 3H, CH_3_), 3.68–3.58 (m, 2H, 5'-H); ^13^C-NMR (DMSO-*d*_6_) 196.5, 161.5, 158.2, 150.3, 136.9, 136.8, 136.7, 135.8, 135.0, 134.8, 133.2, 129.4, 129.1, 128.6, 127.7, 114.2, 113.6, 88.7, 84.8, 73.4, 70.2, 61.2, 55.0, 50.5, 43.4; MS (FAB, Gly) *m/z* 559 (M^+^+H, 29%), 427 (7), 321 (8), 185 (100), 121 (78); HRMS (FAB, Gly) calcd. for C_31_H_30_N_2_O_8_ (M^+^+H): 559.2002; found: 559.2090.

*5-Bis(ethoxycarbonyl)methyl-3-benzyloxymethyluridine* (**8d**): A solution of 5-bromo-3-benzyloxy-methyluridine (**7b**) (500 mg, 1.13 mmol) and diethyl malonate (597 mg, 3.73 mmol) in ethanolic NaOEt [prepared from Na (77.8 mg, 3.38 mmol) in absolute ethanol (10 mL)] was stirred for 24 h at room temperature. The mixture was evaporated under reduced pressure, the residue was dissolved in H_2_O (30 mL), and the mixture was neutralized with NaHSO_4_. The solution was extracted with CHCl_3_ and the extract was dried over MgSO_4_. The solvent was removed under reduced pressure and the residue was purified by column chromatography on silica gel with CHCl_3_–MeOH (150:1) as the eluant to give **8d** (307 mg, 52%) as a colorless oil. ^1^H-NMR (DMSO-*d*_6_) 8.00 (s, 1H, 6-H), 7.34–7.24 (m, 5H, C_6_H_5_), 5.83 (d, *J* = 5.4 Hz, 1H, 1'-H), 5.48 (brd, *J* = 5.4 Hz, 1H, OH), 5.39–5.26 (m, 2H, CH_2_), 5.16–5.14 (m, 1H, OH), 5.04 (brd, *J* = 4.9 Hz, 1H, OH), 4.59 (s, 2H, CH_2_), 4.20–4.10 (m, 5H, CH_2_ × 2 and 2'-H), 4.00–3.98 (m, 1H, 3'-H), 3.89–3.82 (m, 1H, 4'-H), 3.65–3.50 (m, 2H, 5'-H), 1.17 (*J* = 6.9 Hz, 6H, CH_3_ × 2); MS (FAB, NBA) *m/z* 523 (M^+^+H, 32%), 416 (8%), 238 (9), 154 (100), 107 (24); HRMS (FAB, NBA) calcd. for C_24_H_30_N_2_O_11_ (M^+^+H): 523.19343; found: 523.19283.

*5-Bis(methoxycarbonyl)methyl-3-benzyloxymethyl-2'-deoxyuridine* (**8e**). A solution of 5-bromo-3-benzyloxymethyluridine (**7b**) (500 mg, 1.13 mmol) and dimethyl malonate (493 mg, 3.73 mmol) in ethanolic NaOEt [prepared from Na (77.8 mg, 3.38 mmol) in absolute EtOH (10 mL)] was stirred for 24 h at room temperature. The mixture was evaporated under reduced pressure, the residue was dissolved in H_2_O (30 mL), and the mixture was neutralized with NaHSO_4_. The solution was extracted with EtOAc and the extract was dried over MgSO_4_. The solvent was removed under reduced pressure and the residue was purified by column chromatography on silica gel with CHCl_3_–MeOH (150:1) as the eluant to give **8e** (263 mg, 47%) as a colorless oil. ^1^H-NMR (DMSO-*d*_6_) 8.01 (s, 1H, 6-H), 7.35–7.27 (m, 5H, -C_6_H_5_), 5.83 (d, *J* = 4.6 Hz, 1H, 1'-H), 5.47 (brd, *J* = 5.9 Hz, 1H, OH), 5.32 (s, 2H, CH_2_), 5.14–5.12 (m, 1H, OH), 5.05 (brd, *J* = 4.8 Hz, 1H, OH), 4.70 (s, 1H, CH), 4.57 (s, 2H, CH_2_), 4.04–4.02 (m, 1H, 2'-H), 4.01–3.98 (m, 1H, 3'-H), 3.94–3.92 (m, 1H, 4'-H), 3.69 (s, 6H, CH_3_ × 2), 3.68–3.65 (m, 2H, 5'-H); MS (FAB, NBA) *m/z* 495 (M^+^+H, 5%), 238 (10), 176 (8), 154 (100), 85 (47); HRMS (FAB, NBA) calcd. for C_22_H_28_N_2_O_11_ (M^+^+H): 495.15366; found: 494.16221.

*5-(α-Benzoyl)benzyl-3-p-methoxybenzyluridine* (**8f**): A solution of 5-bromo-3-benzyloxymethyluridine (**7b**) (500 mg, 1.13 mmol) and benzylphenylketone (732 mg, 3.73 mmol) in ethanolic NaOEt [prepared from Na (77.8 mg, 3.38 mmol) in absolute ethanol (10 mL)] was stirred for 24 h at room temperature. The mixture was evaporated under reduced pressure, the residue was dissolved in H_2_O (30 mL), and the mixture was neutralized with NaHSO_4_. The solution was extracted with EtOAc and the extract was dried over MgSO_4_. The solvent was removed under reduced pressure and the residue was purified by column chromatography on silica gel with CHCl_3_–MeOH (150:1) as the eluant to give **8f** (297 mg, 47%) as a colorless oil. ^1^H-NMR (DMSO-*d*_6_) 8.01 (d, *J* = 7.7 Hz, 2H, *o*-Bz), 7.57 (t, *J* = 7.7 Hz, 1H, *p*-Bz), 7.48 (t, *J* = 7.2 Hz, *m*-Bz),7.49–7.24 (m, 10H, C_6_H_5_ × 2), 7.10 and 7.04 (each s, total 1H, 6-H), 6.08 (s, 1H, CH), 5.80 (d, *J* = 5.8 Hz, 1H, 1'-H), 5.45 (brd, *J* = 5.8 Hz, 1H, OH), 5.36–5.32 (m, 3H, OH and CH_2_), 5.08 (brd, *J* = 4.8 Hz, 1H, OH), 4.55 (s, 2H, CH_2_), 4.04–3.98 (m, 1H, 2'-H), 3.90–3.88 (m, 1H, 3'-H), 3.76–3.73 (m, 1H, 4'-H), 3.68–3.58 (m, 2H, 5′-H); MS (FAB, NBA) *m/z* 559 (M^+^+H, 5%), 238 (21), 176 (6), 154 (100), 85 (67); HRMS (FAB, NBA) calcd. for C_31_H_30_N_2_O_8_ (M^+^+H): 559.20803; found: 559.20701.

*5-Bis(ethoxycarbonyl)methyl-3-benzyloxymethyl-2'-deoxyuridine* (**8g**). A solution of 5-bromo-3-benzyloxymethyl-2’-deoxyuridine (**7c**) (299 mg, 0.700 mmol) and diethyl malonate (370 mg, 2.31 mmol) in ethanolic NaOEt [prepared from Na (48.3 mg, 2.10 mmol) in absolute EtOH (10 mL)] was stirred for 24 h at room temperature. The mixture was evaporated under reduced pressure, the residue was dissolved in H_2_O (30 mL), and the mixture was neutralized with NaHSO_4_. The solution was extracted with EtOAc and the extract was dried over MgSO_4_. The solvent was removed under reduced pressure and the residue was purified by column chromatography on silica gel with CHCl_3_–MeOH (200:1) as the eluant to give **8g** (170 mg, 48%) as a colorless oil. ^1^H-NMR (CDCl_3_) 8.09 (s, 1H, 6-H), 7.36–7.23 (m, 5H, C_6_H_5_), 6.26 (t, *J* = 6.5 Hz, 1H, 1'-H), 5.47 (s, 2H, CH_2_), 5.09–5.02 (brs, 1H, OH), 4.99–4.93 (brs, 1H, OH), 4.87 (s, 1H, CH), 4.68 (s, 2H, CH_2_), 4.53–4.48 (m, 1H, 3'-H), 4.28–4.16 (m, 4H, CH_2_ × 2), 3.82–3.75 (m, 1H, 4'-H), 3.75–3.54 (m, 2H, 5'-H), 2.47–2.25 (m, 2H, 2'-H), 1.28 (t, *J* = 7.2 Hz, 6H, CH_3_ × 2); MS (FAB, NBA) *m/z* 507 (M^+^+H, 36%), 391 (18), 284 (11), 154 (100), 91 (50); HRMS (FAB, NBA) calcd. for C_24_H_30_N_2_O_10_ (M^+^+H): 507.19783; found: 507.19747.

*5-Bis(methoxycarbonyl)methyl-3-benzyloxymethyl-2'-deoxyuridine* (**8h**). A solution of 5-bromo-3-benzyloxy-2′-deoxymethyluridine (**7c**) (299 mg, 0.700 mmol) and dimethyl malonate (305 mg, 2.31 mmol) in ethanolic NaOEt [prepared from Na (48.3 mg, 2.10 mmol) in absolute EtOH (10 mL)] was stirred for 24 h at room temperature. The mixture was evaporated under reduced pressure, the residue was dissolved in H_2_O (30 mL), and the mixture was neutralized with NaHSO_4_. The solution was extracted with EtOAc and the extract was dried over MgSO_4_. The solvent was removed under reduced pressure and the residue was purified by column chromatography on silica gel with CHCl_3_–MeOH (200:1) as the eluant to give **8h** (141 mg, 42%) as a colorless oil. ^1^H-NMR (CDCl_3_) 8.10 (s, 1H, 6-H), 7.36–7.23 (m, 5H, C_6_H_5_), 6.26 (t, *J* = 6.2 Hz, 1H, 1'-H), 5.47 (s, 2H, CH_2_), 5.10–5.06 (m, 1H, OH), 4.93 (brs, 1H, OH), 4.90 (s, 1H, CH), 4.68 (s, 2H, CH_2_), 4.03–3.98 (m, 1H, 3′-H), 3.93–3.86 (d, *J* = 3.6 Hz, 1H, 4'-H), 3.77 (s, 4H, CH_2_ × 2), 3.58–3.46 (m, 2H, 5'-H), 2.44–2.24 (m, 2H, 2'-H); MS (FAB, NBA) *m/z* 479 (M^+^+H, 5%), 391 (5%), 176 (10), 154 (100), 89 (64); HRMS (FAB, NBA) calcd. for C_22_H_26_N_2_O_10_ (M^+^+H): 479.15875; found: 479.16750.

*5-(α-Benzoyl)benzyl-3-benzyloxymethyl-2'-deoxyuridine* (**8i**). A solution of 5-bromo-3-benzyloxy-2'-deoxymethyl uridine (**7c**) (299 mg, 0.700 mmol) and dimethyl malonate (305 mg, 2.31 mmol) in ethanolic NaOEt [prepared from Na (48.3 mg, 2.10 mmol) in absolute EtOH (10 mL)] was stirred for 24 h at room temperature. The mixture was evaporated under reduced pressure, the residue was dissolved in H_2_O (30 mL), and the mixture was neutralized with NaHSO_4_. The solution was extracted with EtOAc and the extract was dried over MgSO_4_. The solvent was removed under reduced pressure and the residue was purified by column chromatography on silica gel with CHCl_3_–MeOH (200:1) as the eluant to give **8i** (163 mg, 43%) as a colorless oil. ^1^H-NMR (DMSO-*d*_6_) 8.02 (d, *J* = 7.6 Hz, 2H, *O*-Bz), 7.58 (t, *J* = 7.3 Hz, 1H, *p*-Bz), 7.47 (t, *J* = 7.6 Hz, *m*-Bz), 7.40–7.26 (m, 10H, C_6_H_5_ × 2), 7.00 and 6.93 (each s, total 1H, 6-H), 6.12–6.06 (m, 2H, CH and 1′-H), 5.31 (s, 2H, CH_2_), 5.24–5.20 (m, 1H, OH), 4.81-4.77 (m, 1H, OH), 4.56 (s, 2H, CH_2_), 4.05–3.92 (m, 1H, 3'-H), 3.73–3.67 (m, 1H, 4'-H), 3.52–3.35 (m, 2H, 5'-H), 2.17–2.10 (m, 2H, 2'-H); MS (FAB, NBA) *m/z* 543 (M^+^+H, 5%), 282 (21), 238 (11), 154 (100), 107 (20), 85 (44); HRMS (FAB, NBA) calcd. for C_31_H_30_N_2_O_7_ (M^+^+H): 543.20530, found: 543.21377.

*5-Bis(ethoxycarbonyl)methyluridine* (**9a**). (a) A solution of 5-bis(ethoxycarbonyl)methyl-3-*p*-methoxy-benzyluridine (**8a**) (100 mg, 0.191 mmol) and AlCl_3_ (204 mg, 1.53 mmol) in absolute anisole (1.0 mL) was stirred for 12 h at room temperature. To the mixture was added MeOH, and the mixture was evaporated under reduced pressure. The residue was purified by column chromatography on silica gel with CHCl_3_–MeOH (30:1) as the eluant to give **9a** (35.4 mg, 46%) as a colorless oil (Table 3, Entry 1). ^1^H-NMR (DMSO-*d*_6_) 11.58 (s, 1H, 3-NH), 7.87 (s, 1H, 6-H), 5.80 (d, *J* = 5.4 Hz, 1H, 1'-H), 5.40 (brd, *J* = 5.4 Hz, 1H, OH), 5.13–5.09 (m, 1H, OH), 5.02–4.92 (m, 1H, OH), 4.57 (s, 1H, CH), 4.13 (q, *J* = 7.0 Hz, 4H, CH_2_ × 2), 4.01–3.97 (m, 1H, 2'-H), 3.95–3.90 (m, 1H, 3'-H), 3.88–3.82 (m, 1H, 4'-H), 3.60–3.50 (m, 2H, 5'-H), 1.17 (*J* = 7.0 Hz, 6H, CH_3_ × 2). MS (EI) *m/z* 402 (M^+^, 7%), 152 (100); HRMS (EI) calcd. for C_16_H_22_N_2_O_10_ (M^+^) 402.1274; found: 402.1291; (b) A mixture of **8d** (136 mg, 0.260 mmol) and Pd/C (40.8 mg) was stirred at room temperature under an H_2_ atmosphere. After 72 h, the mixture was filtered using a membrane filter (Millex-LH, 0.45 μm), and the filtrate was concentrated *in vacuo*. The residue was purified by column chromatography on silica gel with CHCl_3_–MeOH (30:1) to give **9a** (72.2 mg, 69%) (Table 3, Entry 4).

*5-Bis(methoxycarbonyl)methyluridine* (**9b**). (a) A solution of 5-bis(methoxycarbonyl)methyl-3-*p*-methoxybenzyluridine (**8b**) (100 mg, 0.202 mmol) and AlCl_3_ (216 mg, 1.62 mmol) in absolute anisole (1.0 mL) was stirred for 24 h at room temperature. To the mixture was MeOH, and the mixture was evaporated under reduced pressure. The residue was purified by column chromatography on silica gel with CHCl_3_–MeOH (30:1) as the eluant to give **9b** (31.8 mg, 42%) as a colorless oil (Table 3, Entry 2). ^1^H-NMR (DMSO-*d*_6_) 11.60 (s, 1H, 3-NH), 7.87 (s, 1H, 6-H), 5.79 (d, *J* = 5.6Hz, 1H, 1'-H), 5.39 (brs, 1H, OH), 5.01 (brs, 1H, OH), 5.00 (brs, 1H, OH), 4.62 (s, 1H, CH), 4.03–3.96 (m, 1H, 2'-H), 3.95–3.90 (m, 1H, 3'-H), 3.93–3.84 (m, 1H, 4′-H), 3.66 (s, 6H, CH_3_ × 2), 3.61–3.49 (m, 2H, 5'-H); MS (FAB, Gly) *m/z* 375 (M^+^+H, 3%), 307 (20), 289 (15), 238 (16), 154 (100), 136 (65), 107 (19), 85 (56); HRMS (FAB, Gly) calcd. for C_14_H_19_N_2_O_10_ (M^+^+H): 375.0961; found: 375.1042; (b) A mixture of **8e** (129 mg, 0.26 mmol) and Pd/C (38.7 mg) was stirred at room temperature under an H_2_ atmosphere. After 72 h, the mixture was filtered using a membrane filter (Millex-LH, 0.45 μm), and the filtrate was concentrated *in vacuo*. The residue was purified by column chromatography on silica gel with CHCl_3_/MeOH (30:1) to give **9b** (65.2 mg, 67%) (Table 3, Entry 5).

*5-(α-Benzoyl)benzyluridine* (**9c**). A solution of 5-(α-benzoyl)benzyl-3-*p*-methoxybenzyluridine (**8c**) (100 mg, 0.179 mmol) and AlCl_3_ (191 mg, 1.43 mmol) in absolute anisole (1.0 mL) was stirred for 4 h at room temperature. To the mixture was added MeOH, and the mixture was evaporated under reduced pressure. The residue was purified by column chromatography on silica gel with CHCl_3_–MeOH (30:1) as the eluant to give **9c** (64 mg, 82%) as a pale yellow foam. ^1^H-NMR (DMSO-*d*_6_) 11.50 (s, 1H, 3-NH), 7.97 (d, *J* = 7.8 Hz, 2H, o-Bz), 7.55 (t, *J* = 7.3 Hz, 1H, *p*-Bz), 7.44 (t, *J* = 7.3 Hz, *m*-Bz), 7.33–7.27 (m, 5H, C_6_H_5_), 7.01 and 6.95 (each s, total 1H, 6-H), 6.10 (s, 1H, CH), 5.78–5.72 (m, 1H, 1'-H), 5.37 (brs, 1H, OH), 5.28 (brs, 1H, OH), 5.10–5.06 (m, 1H, OH), 4.68–4.63 (m, 2H. CH_2_), 3.86–3.78 (m, 1H, 2'-H), 3.77–3.71 (m, 1H, 3'-H), 3.66–3.60 (m, 1H, 4'-H), 3.53–3.29 (m, 2H, 5'-H); MS (EI) *m/z* 438 (M^+^, 6%), 306 (17), 201 (55), 158 (13), 115 (10), 105 (100), 77 (25); HRMS (EI) calcd. for C_23_H_22_N_2_O_7_ (M^+^): 438.1427; found: 438.1418.

*5-Bis(ethoxycarbonyl)methyl-2'-deoxyuridine* (**9d**). A mixture of 5-bis(ethoxycarbonyl)methyl-3-benzyloxymethyl-2’-deoxyuridine (**8g**) (50.7 mg 0.100 mmol) and Pd/C (Merck) (15.0 mg) in MeOH (1.0 mL) was stirred under H_2_ atmosphere at room temperature. After 24 h, the mixture was filtered using a membrane filter (Millex-LH, 0.45 μm), and the filtrate was concentrated *in vacuo*. The residue was purified by column chromatography on silica gel with CHCl_3_–MeOH (30:1) as the eluant to give **9d** (24.7 mg, 64%) as a colorless oil. ^1^H-NMR (DMSO-*d*_6_) 11.55 (s, 1H, 3-NH), 7.84 (s, 1H, 6-H), 6.16 (t, *J* = 6.8 Hz, 1H, 1'-H), 5.24 (brd, *J* = 4.1 Hz, 1H, OH), 4.95–4.91 (m, 1H, OH), 4.58 (s, 1H, CH), 4.22–4.18 (m, 1H, 3'-H), 4.06 (q, *J* = 7.2 Hz, 4H, CH_2_ × 2), 3.80–3.78 (m, 1H, 4'-H), 3.55–3.40 (d, *J* = 4.6 Hz, 2H, 5'-H), 2.17–1.97 (m, 2H, 2’-H), 1.17 (t, *J* = 7.1 Hz, 6H, CH_3_ × 2); MS (FAB, NBA) *m/z* 387 (M^+^+H, 9%), 271 (12), 176 (8), 154 (100), 107 (19), 89 (17); HRMS (FAB, NBA) calcd. for C_16_H_22_N_2_O_9_ (M^+^+H): 387.13253; found: 387.14105.

*5-Bis(methoxycarbonyl)methyl-2'-deoxyuridine* (**9e**). A mixture of 5-bis(methoxycarbonyl)methyl-3-benzyloxymethyl-2′-deoxyuridine (**8h**) (50 mg 0.105 mmol) and Pd/C (Merck) (15.0 mg) in MeOH (1.0 mL) was stirred under an H_2_ atmosphere at room temperature. After 72 h, the mixture was filtered using a membrane filter (Millex-LH, 0.45 μm), and the filtrate was concentrated *in vacuo*. The residue was purified by column chromatography on silica gel with CHCl_3_–MeOH (30:1) as the eluant to give **9e** (22.2 mg, 59%) as a colorless oil. ^1^H-NMR (DMSO-*d*_6_) 11.56 (s, 1H, 3-NH), 7.86 (s, 1H, 6-H), 5.81–5.78 (m, 1H, 1'-H), 5.24 (brd, *J* = 4.1 Hz, 1H, OH), 4.95–4.90 (m, 1H, OH), 4.64 (s, 1H, CH), 4.09–4.02 (m, 1H, 3'-H), 3.81–3.76 (m, 1H, 4'-H), 3.66 (s, 4H, CH_2_ × 2), 3.55–3.49 (m, 2H, 5'-H), 2.17–1.99 (m, 2H, 2'-H); MS (FAB, NBA) *m/z* 359 (M^+^+H, 32%), 243 (33%), 154 (100), 107 (10), 85 (62); HRMS (FAB, NBA) calcd. for C_14_H_18_N_2_O_9_ (M^+^+H): 359.10123; found: 359.10827.

*5-(2-Hydroxy-1,2-diphenylethyl)-2'-deoxyuridine* (**13**) *and 5-(1,2-diphenylethyl)-2'-deoxyuridine* (**14**). A mixture of 5-α-benzoylbenzyl-3-benzyloxymethyl-2’-deoxyuridine (**8i**) (81.4 mg 0.150 mmol) and Pd/C (20.3 mg) in MeOH (1.0 mL) was stirred under H_2_ atmosphere at room temperature for 48 h. The mixture was filtered using a membrane filter (Millex-LH, 0.45 μm), and the filtrate was concentrated *in vacuo*. The residue was purified by PTLC with CHCl_3_-MeOH (5:1) as the eluant to give **13** (12.7 mg, 20%) as a light brown oil and **14** (16.5 mg, 27%) as a colorless oil.

**13**: ^1^H-NMR (DMSO-*d*_6_) 11.11 (d, *J* = 10.6 Hz, 1H, 3-NH), 7.97 and 7.90 (each s, 1H, 6-H), 7.25–7.10 (m, 10H, C_6_H_5_ × 2), 6.11–6.05 (m, 1H, 1'-H), 5.32–5.09 (m, 4H, CH × 2 and OH × 2), 4.53 (s, 1H, OH), 4.30–4.22 (m, 1H, 3'-H), 3.82–3.78 (m, 1H, 4'-H), 3.70–3.59 (m, 2H, 5'-H), 2.11–1.90 (m, 2H, 2'-H); MS (FAB, NBA) *m/z* 425 (M^+^+H, 19%), 291 (65), 202 (17), 176 (8), 154 (100), 107 (26), 77 (21); HRMS (FAB, NBA) calcd. for C_23_H_24_N_2_O_6_ (M^+^+H): 425.16344; found: 425.17190.

**14**: ^1^H-NMR (DMSO-*d*_6_) 11.19 (s, 1H, 3-NH), 8.04 and 8.00 (each s, 1H, 6-H), 7.25–7.10 (m, 10H, C_6_H_5_ × 2), 6.16 (t, *J* = 6.6 Hz, 1H, 1'-H), 5.32–5.20 (m, 4H, CH_2_ and OH × 2), 5.24–5.20 (m, 1H, OH), 4.32–4.10 (m, 2H, 3'-H and CH), 3.84–3.80 (m, 1H, 4'-H), 3.68–3.60 (m, 2H, 5'-H), 2.12–2.03 (m, 2H, 2'-H); MS (FAB, NBA) *m/z* 409 (M^+^+1, 6%), 154 (100), 136 (60), 107 (16), 89 (14); HRMS (FAB, NBA) calcd. for C_23_H_24_N_2_O_5_ (M^+^+H): 409.16852; found: 409.17779.

*Pd/C-catalyzed hydrogenation of 5-(α-benzoyl)benzyl-3-p-methoxybenzyluridine* (**8c**) (Scheme 7). A mixture of **8c** (100 mg, 0.179 mmol) and Pd/C (30.0 mg) was stirred at room temperature under an H_2_ atmosphere. After 48 h, the mixture was filtered using a membrane filter (Millex-LH, 0.45 μm), and the filtrate was concentrated *in vacuo*. The residue was purified by column chromatography on silica gel with CHCl_3_/MeOH (100:1 to 50:1) to give 3-*p*-methoxybenzyl-5-(1,2-diphenylethyl)uridine (**10**, 43.9 mg, 45%) and 3-*p*-methoxybenzyl-5-(2-hydroxy-1,2-diphenylethyl)uridine (**11**, 34.1 mg, 34%). 

*3-p-Methoxybenzyl-5-(1*,*2-diphenylethyl)uridine* (**10**). A colorless foam. ^1^H-NMR (DMSO-*d*_6_) 8.26 and 8.20 (each s, 1H, 6-H), 7.24–7.18 (m, 12H, C_6_H_5_ × 2 and *o*-PMB), 6.80 (d, *J* = 6.8 Hz, *m*-PMB), 5.81 (d, *J* = 4.1 Hz, 1H, 1'-H), 5.48–5.39 (m, 4H, OH × 2 and CH_2_), 5.12–5.08 (m, 1H, OH), 4.89 (s, 2H, CH_2_) 4.27–4.21 (m, 1H, 2'-H), 4.09–4.00 (m, 2H, CH and 3'-H), 3.93–3.90 (m, 1H, 4'-H), 3.69 (s, 3H, CH_3_), 3.40–3.35 (m, 2H, 5'-H); MS (EI) *m/z* 544 (M^+^, 2%), 453 (16), 321 (86), 121 (100), 91 (8); HRMS (EI) calcd. for C_31_H_32_N_2_O_7_ (M^+^): 544.2210; found: 544.2197.

*3-p-Methoxybenzyl-5-(2-hydroxy-1*,*2-diphenylethyl)uridine* (**11**). Colorless oil. ^1^H-NMR (DMSO*-d_6_*) 8.26 and 8.19 (each s, 1H, 6-H), 7.34–7.04 (m, 12H, C_6_H_5_ × 2 and *o*-PMB), 6.80 (d, *J*= 6.8 Hz, *m*-PMB), 5.81 (d, *J*= 3.9 Hz, 1H, 1'-H), 5.46 (brs, 1H, OH), 5.41 (brs, 1H, OH), 5.14–5.08 (m, 1H, OH), 4.82 (s, 2H, CH_2_), 4.27–4.21 (m, 1H, 2'-H), 4.09–4.03 (m, 2H, CH and 3'-H), 3.93–3.90 (m, 1H, 4'-H), 3.76–3.60 (m, 5H, CH and OH and CH_3_), 3.40–3.34 (m, 2H, 5'-H); MS (FAB, Gly) *m/z* 561 (M^+^+H, 13%), 543 (7), 369 (5), 277 (14), 185 (100), 121 (33); HRMS (FAB, Gly) calcd. for C_31_H_32_N_2_O_8_ (M^+^+H): 561.2159; found: 561.2228.

*Pd/C(en)-catalyzed hydrogenation of 5-(α-Benzoyl)benzyl-3-p-methoxybenzyluridine* (**8c**) (Scheme 7). A mixture of **8c** (100 mg, 0.179 mmol) and 10% Pd/C(en) (30.0 mg) was stirred at room temperature under an H_2_ atmosphere. After 48 h, the mixture was filtered using a membrane filter (Millex-LH, 0.45 μm), and the filtrate was concentrated *in vacuo*. The residue was purified by column chromatography on silica gel with CHCl_3_–MeOH (100:1 to 50:1) to give **11** (63.6 mg, 63%) as a colorless oil.

*5-(1*,*2-Diphenylethyl)uridine* (**12**). According to the procedure for the removal of the PMB group of 5-bis(ethoxycarbonyl)methyluridine (**8a**), **12** (61 mg, 79%) was obtained as a colorless foam. ^1^H-NMR (DMSO-*d*_6_) 11.23 (s, 1H, 3-NH), 8.14 and 8.10 (each s, 1H, 6-H), 7.24–7.18 (m, 10H, C_6_H_5_ × 2), 5.77 (d, *J* = 3.4 Hz, 1H, 1'-H), 5.43 (d, *J* = 6.2 Hz, 2H, CH_2_), 5.36 (brs, 1H, OH), 5.36 (brs, 1H, OH), 5.12 (brs, 1H, OH), 4.24–4.10 (m, 3H, 2'-H and 3’-H and CH), 3.72–3.60 (m, 3H, 4'-H and 5'-H); MS (FAB, NBA) *m/z* 425 (M^+^+H, 21%), 329 (8), 154 (100), 136 (69), 89 (21); HRMS (FAB, NBA) calcd. for C_23_H_24_N_2_O_6_ (M^+^+H): 425.1634; found: 425.1705.

*2*,*4-Dimethyl-7-ethoxycarbonyl-7-phenyl-2,4-diazabicyclo[4,1,0]heptane-3*,*5-dione* (**15a**). (a) 5-Bromo-1,3-dimethyluracil (**1a**, 657 mg, 3.00 mmol) was added to a stirred solution of ethyl phenylacetate (1.63 g, 9.90 mmol) in ethanolic NaOEt [prepared from Na (207 mg, 9.00 mmol) in absolute EtOH (30 mL)] and the mixture was stirred at room temperature for 10 h. The solvent was removed under reduced pressure, and the residue was dissolved in H_2_O (20 mL). The solution was neutralized with c.HCl, and the mixture was extracted with CHCl_3_. The extract was dried over MgSO_4_, and concentrated *in vacuo*. The residue was purified by column chromatography on silica gel with CHCl_3_ as the eluant and recrystallized from Et_2_O to give **15a** (589 mg, 65%). m.p. 123.5–125 °C; UV λ_max._ (EtOH) only end absorption; ^1^H-NMR (CDCl_3_) 7.48–6.98 (m, 5H, Ph), 4.15 (q, *J* = 7.0 Hz, 2H, CH_2_), 3.76 and 3.22 (each d, each *J* = 9.0 Hz, each 1H, 5 and 6-H), 3.25 and 2.76 (each s, each 3H, NMe), 1.18 (t, *J* = 7 Hz, 3H, CMe); ^13^C-NMR (CDCl_3_) 170.5, 165.6, 150.8, 130.7, 129.2, 128.8, 128.8, 62.3, 45.1, 36.3, 30.3, 27.2, 14.0; MS (EI) *m/z* 302 (M^+^, 13%), 303 (13), 256 (100), 228 (50), 227 (24), 199 (23) 143 (27); Anal calcd. for C_16_H_18_N_2_O_4_: C, 63.56; H, 6.00; N, 9.27%; found: C, 63.28; H, 6.00; N, 9.16; (b) 5-Bromo-1,3-dimethyluracil (**1a**) (657 mg, 3.00 mmol) was dissolved in a solution of ethyl phenylacetate (1.63 g, 9.90 mmol) and DBU (1.37 g, 9.00 mmol) in anhydrous DMF (30 mL). The mixture was stirred at room temperature for 4 days and then the solvent was removed under reduced pressure. The residue was treated as described above to give **15a** (635 mg, 70%), which was identical to the product obtained above.

*7-Cyano-1,4-dimethyl-7-phenyl-2,4-diazabicyclo[4,1,0]heptane-3,5-dione* (**15b**). 5-Bromo-1,3-dimethyluracil (**1a**, 657 mg, 3.00 mmol) was added to a stirred solution of benzylcyanide (1.16 g, 9.90 mmol) in ethanolic NaOEt [prepared from Na (207 mg, 9.00 mmol) in absolute EtOH (30 mL)] and the mixture was stirred at room temperature for 10 min. The mixture was neutralized with Amberlite CG-50 (H^+^) and filtered. The ion exchanger was washed with EtOH. The combined filtrates were concentrated under reduced pressure, and the residue was purified by column chromatography on silica gel with chloroform as the eluant to give **15b** (720 mg, 94%), which was recrystallized from CHCl_3_–Et_2_O. m.p. 134–135 °C; UV λ_max._ (EtOH) only end absorption; ν_max_ 2240 cm^–1^ (CN); ^1^H-NMR (CDCl_3_) 7.68–7.06 (5H, m, Ph), 3.57 (1H, d, *J* = 8.5 Hz, 6-H), 3.27 and 3.24 (each 3H, each s, NMe), 3.02 (d, *J* = 8.5 Hz, 1H, 5-H); ^13^C-NMR (CDCl_3_) 162.9, 150.7, 131.3, 129.5, 129.1, 125.9, 114.7, 46.5, 35.6, 31.4, 28.0, 25.0; MS (EI), *m/z* 255 (M^+^, 70%), 198 (36), 170 (100), 169 (85); Anal calcd. for C_14_H_13_N_3_O_4_: C, 65.87; H, 5.13; N, 16.46%; found: C, 65.88; H, 5.12; N, 16.51.

*2,4-Dimethyl-7,9-diphenyl-2,4-diazabicyclo[4,3,0]nonane-3,5,8-trione* (**16**). 5-Bromo-1,3-dimethyl-uracil (**1a**) (657 mg, 3.00 mmol) was added to a stirred solution of dibenzylketone (2.08 g, 9.90 mmol) in ethanolic NaOEt [prepared from Na (207 g, 9.00 mmol) in absolute EtOH (30 mL)] and the mixture was stirred at room temperature for 30 min. The mixture was neutralized with Amberlite CG-50 (H^+^) and filtered. The ion exchange resin was washed with EtOH. The combined filtrates were concentrated under reduced pressure, and the residue was purified by chromatography on silica gel with CHCl_3_ as the eluant to give **16** (638 mg, 61%), which was recrystallized from chloroform-ether. m.p. 220–221 °C; UV λ_max._ (EtOH) 266 nm (ε 25,100 dm^3^mol^–1^cm^–1^); *m/z* 348 (M^+^, 52%), 208 (28), 180 (100); ^1^H-NMR (CDCl_3_) 7.70–7.07 (m, 10H, Ph), 5.06 (d, *J* = 8.0 Hz, 1H, 4a or 7a-H), 4.93 (d, *J* = 2.0 Hz, 1H, 5 or 7-H), 4.13 (dd, *J* = 8.5 Hz and 8.0 Hz, 1H, 4a or 7a-H), 3.95 (dd, *J* = 8.5, 2.0 Hz, 1H, 5 or 7-H), 2.73 and 3.29 (each s, each 3H, NMe); ^13^C-NMR (CDCl_3_) 166.4, 155.7, 151.2, 137.5, 134.7, 129.3, 128.6, 128.3, 128.3, 128.1, 126.1, 103.1, 73.3, 64.0, 54.4, 35.8, 28.2; Anal calcd. for C_21_H_20_N_2_O_3_: C, 72.39; H, 5.79; N, 8.04%; found: C, 72.14; H, 5.87; N, 8.01.

*1,3-Dimethyluracil-6-(α-acetyl)acetic acid ethyl ester* (**17**). (a) A solution of 5-bromo-1,3-dimethyluracil (**1a**, 2.20 g, 10.0 mmol) and ethyl acetoacetate (4.32 g, 33.0 mmol) in ethanolic NaOEt [prepared from Na (690 mg, 30.0 mmol) in absolute EtOH (100 mL)] was stirred for 3 days at room temperature. The mixture was evaporated under reduced pressure, and the residue was treated with H_2_O. The resulting precipitate was filtered off, and the mother liquor was extracted with CHCl_3_. The extract was dried over Na_2_SO_4_, and the solvent was removed under reduced pressure. The residue was treated with Et_2_O and the resulting precipitate was filtered off. The combined precipitate was washed with Et_2_O to afford the recovered (**1a**) (700 mg, 32%), which was identical to the authentic sample. The water layer was neutralized with c.HCl, and the mixture was extracted with CHCl_3_. The extract was concentrated *in vacuo*, and the residue was purified by column chromatography on silica gel with CHCl_3_ as the eluant to give **17** (1.66 g, 62%). m.p. 100–103 °C; UV λ_max_(EtOH) 268 nm (ε 12,400 dm^3^mol^–1^cm^–1^); ^1^H-NMR (CDCl_3_) 13.10 (s, CH, 1H, deuterium exchangeable), 5.68 (s, 1H, 5-H), 4.32 (q, *J* = 7.5 Hz, 2H, CH_2_), 3.37 and 3.26 (each s, each 3H, NMe), 2.04 (s, 3H, CMe), 1.28 (t, *J* = 7.5 Hz, 3H, CMe); MS (EI) *m/z* 268 (M^+^, 74%), 222 (32), 207 (54), 82 (32), 43 (100); Anal. calcd. for C_12_H_16_N_2_O_5_: C, 53.72; H, 6.01; N, 10.44%; found: C, 53.48; H, 6.12; N, 10.52; (b) To a stirred solution of 6-chloro-1,3-dimethyluracil (**19**) (349 mg, 2.00 mmol) and ethyl acetoacetate (859 mg, 6.60 mmol) in anhydrous DMF (5 mL) was added sodium hydride (60% in mineral oil) (240 mg, 6.00 mmol). The mixture was stirred at room temperature for 5 days, and the solvent was removed under reduced pressure. The residue was dissolved in H_2_O (20 mL) and then washed with CHCl_3_. The aqueous layer was neutralized with conc. HCl and extracted with CHCl_3_. The extract was dried over Na_2_SO_4_, and the solvent was removed under reduced pressure. The residue was purified by column chromatography on silica gel with CHCl_3_ as the eluant to give **17** (311 mg, 58%), which was identical to the sample prepared above.

*1*,*3*,*6-Trimethyluracil* (**18**) (CAS: 13509-52-9). A mixture of the 1,3-dimethyluracil-6-(α-acetyl)acetic acid ethyl ester (**2**, 1.32 g, 4.92 mmol) and hydrobromic acid (47%) was refluxed for 1 h. The solvent was removed under reduced pressure, and the residue was treated with H_2_O (30 mL). The suspension was extracted with CHCl_3_, and the extract was dried over Na_2_SO_4_. The solvent was removed under reduced pressure to give **18** (743 mg, 98%), which was identical to the authentic sample.

*3-p-Methoxybenzyluridine-6-(α-acetyl)acetic acid ethyl ester* (**20**). To a stirred solution of 5-bromo-3-*p*-methoxybenzyluridine (**7a**, 1.33 g, 3.00 mmol) and ethyl acetoacetate (1.29 g, 9.90 mmol) in anhydrous DMF (30 mL) was added potassium *t*-butoxide (1.01 g, 9.00 mmol). The mixture was stirred at room temperature for 3 days, and the solvent was removed under reduced pressure. The residue was dissolved in H_2_O (10 mL) and then washed with CHCl_3_. The aqueous layer was neutralized with concentrated NaHSO_4_ and extracted with CHCl_3_. The extract was dried over Na_2_SO_4_, and the solvent was removed under reduced pressure. The residue was purified by column chromatography on silica gel with CHCl_3_–MeOH (150:1) as the eluant to give **20** as a light brown foam (946 mg, 64%). ^1^H-NMR (CDCl_3_) 13.19 (s, 1H, CH), 7.43 and 6.84 (each d, each *J* = 7.8 Hz, each 2H, C_6_H_4_), 5.67 (d, 1H, *J* = 3.4 Hz, 5-H), 5.39 (d, *J* = 5.4 Hz, 1H, 1′-H), 5.37–5.00 (m, 4H, OH × 2, CH_2_), 4.90 (brd, *J* = 3.9 Hz, 1H, OH), 4.21–4.16 (m, 3H, 2'-H, CH_2_), 3.88 (m, 1H, 3'-H), 3.78 (s, 3H, CH_3_), 3.72 (m, 1H, 4'-H), 3.48 (m, 2H, 5'-H), 2.07 and 2.00 (each s, total 3H, CH_3_), 1.22 (brt, *J* = 7.1 Hz, 3H, CH_3_). MS (EI) *m/z* 492 (M^+^, 8%), 360 (32), 314 (18), 162 (15), 121 (100). HRMS (EI) calcd. for C_23_H_28_N_2_O_10_ (M^+^): 492.1744; found: 492.1756.

*3-Benzyloxymethyluridine-6-(α-acetyl)acetic acid ethyl ester* (**21a**). To a stirred solution of 5-bromo-3-benzyloxymethyluridine (**7b**, 452 mg, 1.02 mmol) and ethyl acetoacetate (586 mg, 4.50 mmol) in anhydrous DMF (30 mL) was added potassium *t*-butoxide (337 mg 3.00 mmol). The mixture was stirred at room temperature for 3 days and the solvent was removed under reduced pressure. The residue was dissolved in H_2_O (10 mL) and then washed with CHCl_3_. The aqueous layer was neutralized with concentrated NaHSO_4_ and extracted with CHCl_3_. The extract was dried over Na_2_SO_4_ and the solvent was removed under reduced pressure. The residue was purified by column chromatography on silica gel with CHCl_3_–MeOH (200:1) as the eluant to give **21a** (242 mg, 48%) as a colorless oil. ^1^H-NMR (CDCl_3_) 12.91 (s, 1H, CH), 7. 31 (m, 5H, C_6_H_5_), 5.75 (d, 1H, *J* = 3.1 Hz, 5-H), 5.54 (d, *J* = 5.9 Hz, 1H, 1'-H), 5.30 (s, 2H, CH_2_), 5.22 (brd, *J* = 4.4 Hz, 1H, OH), 5.10–5.07 (m, 1H, OH), 4.99–4.96 (m, 1H, OH), 4.61 (s, 2H, CH_2_), 4.21–4.15 (m, 3H, 2’-H and CH_2_), 4.02-3.99 (m, 1H, 3'-H), 3.62–3.58 (m, 1H, 4'-H), 3.49–3.39 (m, 2H, 5'-H), 2.01 and 1.97 (each s, total 3H, CH_3_), 1.17 (brt, *J* = 7.1 Hz, 3H, CH_3_); ^13^C-NMR (CDCl_3_) 177.3, 169.7, 161.9, 151.7, 128.4, 127.8, 127.5, 106.5, 106.2, 97.3, 94.7, 93.6, 84.2, 72.6, 72.5, 70.5, 68.9, 62.4, 61.7, 20.18, 14.0; MS (FAB, NBA) *m/z* 493 (M^+^+H, 12%), 361 (10), 331 (11), 154 (100), 91 (41); HRMS (FAB, NBA) calcd. for C_23_H_28_N_2_O_10_ (M^+^+H) 493.18218; found: 493.18141.

*3-Benzyloxymethyl-2'-deoxyuridine-6-(α-acetyl)acetic acid ethyl ester* (**21b**). To a stirred solution of 5-bromo-3-*p*-methoxybenzyl-2'-deoxyuridine (**7c**, 297 mg, 0.700 mmol) and ethyl acetoacetate (0.290 mL, 2.32 mmol) in anhydrous DMF (5 mL) was added potassium *t*-butoxide (237 mg, 2.11 mmol). The mixture was stirred at room temperature for 3 days, and the solvent was removed under reduced pressure. The residue was dissolved in H_2_O (10 mL) and then washed with CHCl_3_. The aqueous layer was neutralized with concentrated NaHSO_4_ and extracted with CHCl_3_. The extract was dried over Na_2_SO_4 _and the solvent was removed under reduced pressure. The residue was purified by chromatography on silica gel with CHCl_3_–MeOH (200:1) as the eluant to give **21b** (141 mg, 42%) as a colorless oil. ^1^H-NMR (CDCl_3_) 13.09 (s, 1H, CH), 7.39–7.29 (m, 5H, C_6_H_5_), 5.82–5.76 (m, 1H, 5-H), 5.63–5.60 (m, 1H, 1'-H), 5.43 (s, 2H, CH_2_), 5.38 (brs, 1H, OH), 4.96 (brs, 1H, OH), 4.74 (s, 2H, CH_2_), 4.27 (q, *J* = 7.1 Hz, 2H, CH_3_), 3.92–3.86 (m, 1H, 3'-H), 3.80–3.74 (m, 1H, 4'-H), 3.62–3.40 (m, 2H, 5'-H), 2.08–1.96 (m, 5H, CH_3_ and 2'-H), 1.29 (brt, *J* = 7.0 Hz, 3H, CH_3_); MS (FAB, NBA) *m/z* 477 (M^+^+H, 9%), 361 (29), 256 (6), 154 (100), 91 (31); HRMS (FAB, NBA) calcd. for C_23_H_28_N_2_O_9_ (M^+^+H): 477.18728; found: 477.18841.

*Uridine-6-(α-acetyl)acetic acid ethyl ester* (**22a**). A mixture of 3-benzyloxymethyluridine-6-(α-acetyl)acetic acid ethyl ester (**21a**, 100 mg, 0.203 mmol) and Pd/C (30.0 mg) in MeOH (1 mL) was stirred under H_2_ atmosphere at room temperature for 24 h. The mixture was filtered using a membrane filter (Millex-LH, 0.45 μm), and the filtrate was concentrated *in vacuo*. The residue was purified by column chromatography on silica gel with CHCl_3_–MeOH (40:1) as the eluant to give uridine-6-acetoacetic acid ethyl ester (**22a**, 59.0 mg, 78%) as a light brown oil. ^1^H-NMR (CDCl_3_) 12.89 (s, 1H, CH), 11.42 (s, 1H, 3-NH), 5.66 (d, *J* = 2.2 Hz, 1H, 5-H), 5.64 (d, *J* = 5.9 Hz, 1H, 1'-H), 5.24–5.18 (m, 1H, OH), 5.12–5.07 (m, 1H, OH), 5.13–4.97 (m, 1H, OH), 4.17–4.11 (m, 1H, 2'-H), 4.10–3.57 (m, 6H, CH_2_, 3'-H, 4'-H and 5'-H), 2.02 and 1.98 (each s, total 3H, CH_3_), 1.18 (brt, *J* = 7.1 Hz, 3H, CH_3_); ^13^C-NMR (CDCl_3_) 176.5, 175.8, 169.5, 162.5, 105.8, 97.5, 93.8, 92.9, 84.4, 72.2, 70.1, 62.1, 61.2, 19.8, 13.7; MS (FAB, Gly) *m/z* 373 (M^+^+H, 5%), 277 (10), 270 (33), 184 (100), 115 (57); HRMS (FAB, Gly) calcd. for C_15_H_20_N_2_O_9_ (M^+^+H): 373.1169; found: 373.1251.

*2'-Deoxyuridine-6-(α-acetyl)acetic acid ethyl ester* (**22b**). A mixture of 3-benzyloxymethyl-2'-deoxyuridine-6-(α-acetyl)acetic acid ethyl ester (**21b**, 100 mg, 0.210 mmol) and Pd/C (30.0 mg) in MeOH (1 mL) was stirred under H_2_ atmosphere at room temperature for 48 h. The mixture was filtered using a membrane filter (Millex-LH, 0.45 μm), and the filtrate was concentrated *in vacuo*. The residue was purified by column chromatography on silica gel with CHCl_3_–MeOH (50:1) as the eluant to give **22b** (70.3 mg, 94%) as a colorless foam. ^1^H-NMR (CDCl_3_) 12.83 (s, 1H, CH), 11.35 (s, 1H, 3-NH), 5.73–5.70 (m, 1H, 5-H), 5.58–5.56 (m, 1H, 1'-H), 5.08–4.90 (m, 1H, OH), 4.54–4.48 (m, 1H, OH), 4.23–4.10 (m, 3H, CH_2_ and 3'-H), 3.61–3.44 (m, 3H, 4'-H and 5'-H), 2.02–1.88 (m, 3H, CH_3_), 1.23–1.06 (m, 3H, CH_3_); MS (FAB, NBA) *m/z* 356 (M^+^+H, 9%), 241 (39), 195 (17), 154 (100); HRMS (FAB, NBA) calcd. for C_15_H_20_N_2_O_8_ (M^+^+H): 357.12978; found: 357.13060.

## 4. Conclusions

We have accounted for the diversity of the C–C bond formation reaction between 5-halogenouracil or 5-halogenouridine derivatives **1** and **7** and carbanions. The reactions of 5-halogenouracil and 5-halogenouridine derivatives **1** and **7** with active methylene compounds under basic conditions selectively gave 5-substituted uracil derivatives **2**, **5** and **8** via the isolable 5,6-disubstituted 5,6-dihydrouracil derivatives **4**, 4-diazabicyclo[4.1.0]heptane derivatives and 4-diazabicyclo[4.1.0]nonane **15 **and **16** and 6-substituted uracil and uridine derivatives **17**, **20** and **21**, all of which were extremely dependent on the nature of the carbanions.
